# Developing a Social, Cultural and Economic Report Card for a Regional Industrial Harbour

**DOI:** 10.1371/journal.pone.0148271

**Published:** 2016-02-03

**Authors:** Sean Pascoe, Renae Tobin, Jill Windle, Toni Cannard, Nadine Marshall, Zobaidul Kabir, Nicole Flint

**Affiliations:** 1 CSIRO Oceans and Atmosphere, Brisbane, Queensland, Australia; 2 Centre for Sustainable Tropical Fisheries and Aquaculture and the College of Marine and Environmental Sciences, James Cook University, Townsville, Queensland, Australia; 3 School of Business and Law, Central Queensland University, Rockhampton, Queensland, Australia; 4 CSIRO Land and Water, Townsville, Queensland, Australia; University of Auckland, NEW ZEALAND

## Abstract

Report cards are increasingly used to provide ongoing snap-shots of progress towards specific ecosystem health goals, particularly in coastal regions where planners need to balance competing demands for coastal resources from a range of industries. While most previous report cards focus on the biophysical components of the system, there is a growing interest in including the social and economic implications of ecosystem management to provide a greater social-ecological system understanding. Such a report card was requested on the Gladstone Harbour area in central Queensland, Australia. Gladstone Harbour adjoins the southern Great Barrier Reef, and is also a major industrial and shipping port. Balancing social, economic and environmental interests is therefore of great concern to the regional managers. While environmental benchmarking procedures are well established within Australia (and elsewhere), a method for assessing social and economic performance of coastal management is generally lacking. The key aim of this study was to develop and pilot a system for the development of a report card relating to appropriate cultural, social and economic objectives. The approach developed uses a range of multicriteria decision analysis methods to assess and combine different qualitative and quantitative measures, including the use of Bayesian Belief Networks to combine the different measures and provide an overall quantitative score for each of the key management objectives. The approach developed is readily transferable for purposes of similar assessments in other regions.

## Introduction

Report cards are used in a wide range of areas as a communication tool to inform key stakeholders about the relative performance of an industry or activity. Originating in schools as a means of informing parents about students’ progress, they have evolved to report on the relative performance of schools, universities and health care to provide an incentive for these industries to improve their performance [1–3]. Variations of the report card system have also been applied to the food industry to inform consumers about healthy eating choices and/or sustainability of the product [4, 5], and has been extended to assessing relative regional economic performance of local governments [6].

The use of report cards to provide snap-shots of progress towards the achievement of specific ecosystem health goals has become increasingly popular since the 1990s [7]. Within Australia, environmental based report cards have been developed for the Great Barrier Reef, the Fitzroy Basin, Tamar Valley and south east Queensland [7]. These report cards generally focus on the environmental performance directly, based on monitoring a range of environmental indicators and assessing these against some preferred state. In coastal, estuarine and marine settings, report cards provide a succinct way to communicate science based on monitoring, both ambient and event-based, and associated uncertainty [7–9]. Further, they provide a mechanism to communicate science across a wide range of scientific disciplines and potentially allow for the context of time and place to be incorporated [10].

Most environmentally-oriented report cards are focused on the biophysical components of the system. However, there is growing interest in ecosystem based management (EBM) approaches, and the corresponding concepts of ecologically sustainable development (ESD), both of which also consider the economic and social implications of ecosystem management [11].

A key challenge of developing a social, economic and cultural report card is the lack of well-defined benchmarks, lack of well-defined indicators and the often intangibility of the indicators that are identified. Environmental report cards are generally based on clearly identifiable indicators that may be objectively measured and generally with established threshold limits. For example, water quality can be accessed through measuring the presence of heavy metals, pesticides or other chemicals in the water, nitrogen level and suspended solids in the water. Acceptable threshold levels for acceptable levels of these indicators are generally established a priori [12], and water quality can be assessed as acceptable if above the threshold or unacceptable if below.

In contrast, social wellbeing is a fairly abstract concept, and not readily observable. The relationship between different indicators, such as satisfaction levels, and overall acceptable levels is generally undefined [13]. Trade-offs between, and additionality of, these indicators is also generally not quantitatively considered where social impact assessments have been performed. Instead, outcomes are reported as a series of qualitative indicators. Similarly, economic indicators, such as gross value of production, are also often driven by a range of external factors (e.g., exchange rates), so defining thresholds relevant to regional coastal management is also complex.

The aim of this paper is to present, by way of example, an approach to develop a social, cultural and economic report card to assess the performance of regional management of an industrial region in a highly sensitive environment. Such a report card was requested by the Gladstone Healthy Harbour Partnership (GHHP), a consortium of local and state government bodies as well as key industry bodies with the combined objective of ensuring the sustainability of the region from a social, economic and environmental perspective. While some of the measures and indicators presented are specific to the Gladstone Harbour case study, the general approach is more broadly applicable. For this reason, some of the specific details on survey results will be glossed over, with the emphasis instead being placed on how the process was undertaken. Full details on all the measures, data and full results are provided in Pascoe et al [14].

## The Gladstone Harbour Region

**Fig 1:**
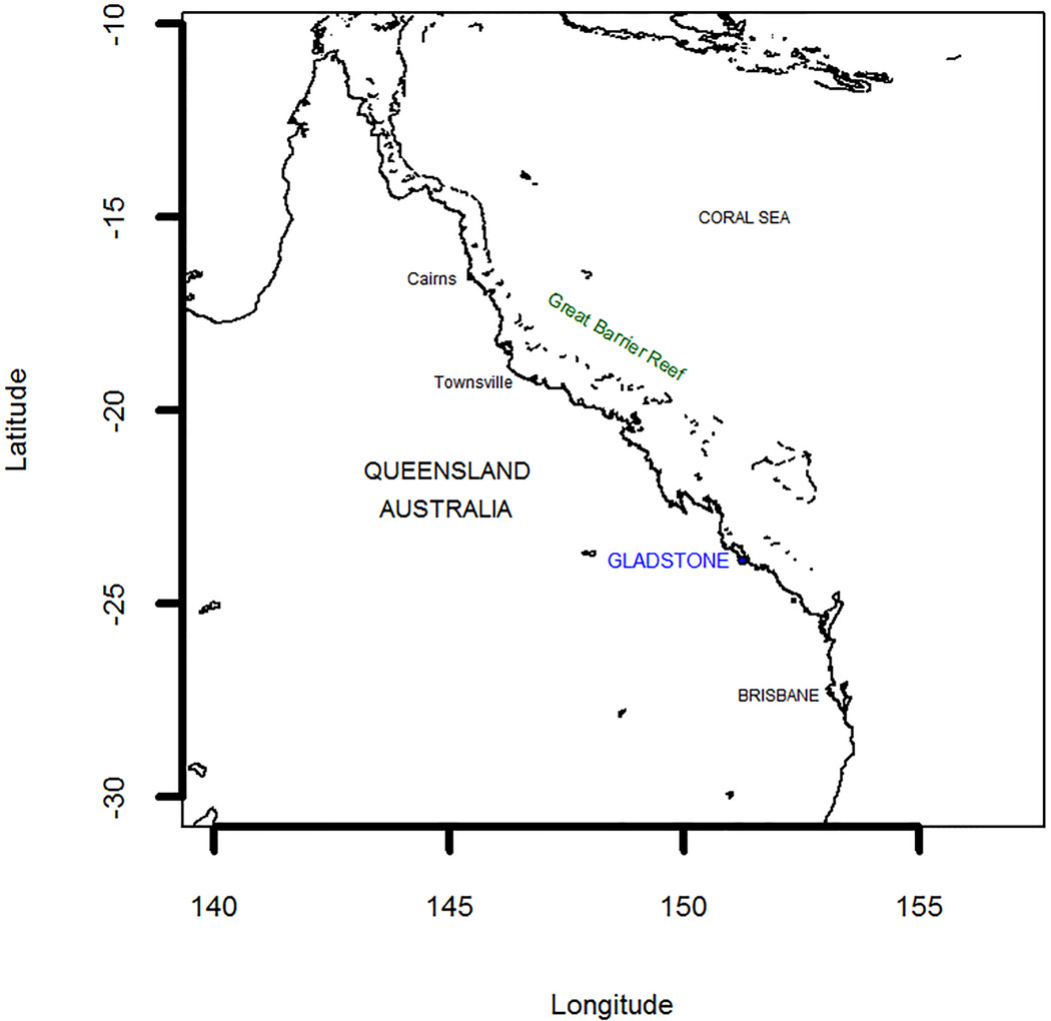
Location of Gladstone Harbour.

Gladstone is located on the Queensland central coast, and is a key access point to the southern Great Barrier Reef (Fig 1). Historically an agricultural area, it has developed over the last 30 years into a major industrial hub centred on the Port of Gladstone. Shipping in the region brings in key inputs for the industrial sectors (e.g., bauxite for the two aluminium smelters) as well as exports from these industries (particularly cement and alumina). It is also a key port for coal exports, with roughly 70 per cent of the shipping activity in 2012–13 involving coal exports [15]. Gladstone is the third largest coal exporting port in Australia, and the fourth largest in the world. The first of three LNG (liquefied natural gas) processing plants in the region also commenced operation in 2014, with this sector expected to expand shipping activity further in the near future. To facilitate the industrial development in the region, the harbour has undergone a series of dredging programs to allow more and bigger ships into the harbour. At-sea dumping of the dredge spoils has been controversial, particularly given the proximity of the harbour to the Great Barrier Reef Marine Park.

The GHHP was established in 2013 to provide support for improved decision making with respect to environmental management of Gladstone Harbour [16]. To assist in this decision making, the GHHP proposed the development of an annual “report card” on the health of the Harbour, and to identify priorities for future improvements and/or restoration projects. GHHP had previously commissioned several studies to determine the appropriate operational objectives under each of the broader social, cultural and economic domains, as well as the key components and indicators for measuring the success of these objectives [16, 17].

## Methods

Multicriteria approaches have been found to be useful for integrating social impacts with other measures [18]. These provide a formalised structure to aggregate a wide range of different measures, many of which are qualitative in nature. Such an approach was adopted as the general framework for the development of the report card.

**Fig 2:**
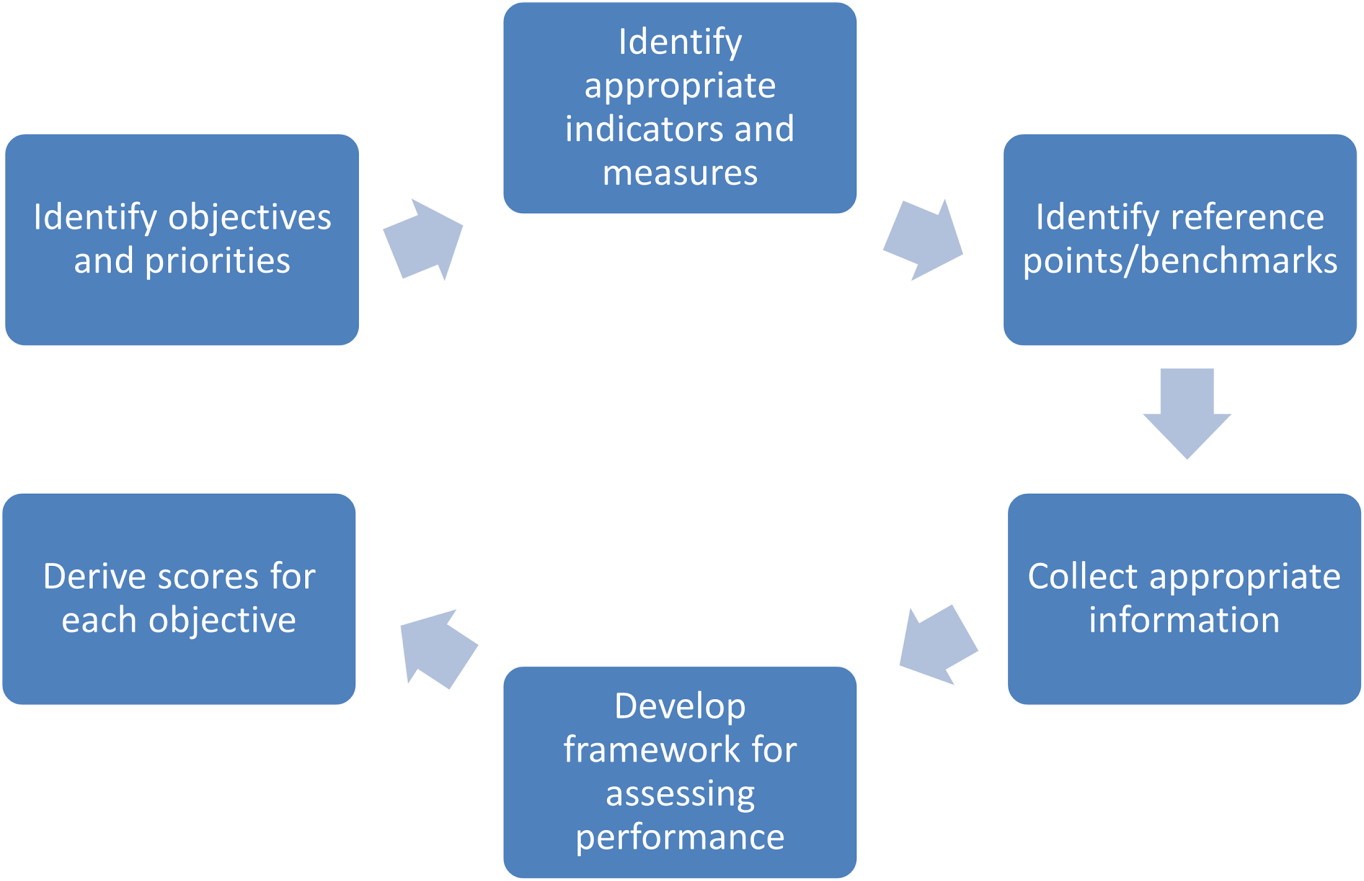
Logical steps for report card development.

The development of a successful report card involves a series of logical steps [19], summarised in Fig 2. First, the objectives of the management against which performance is measured needs to be established. Second, indicators and measures need to be determined that reflect the performance against these objectives, and targets or benchmarks also need to be established for these indicators. Once data are collected on these indicators, a means of assessing overall performance, taking into account the relative importance of the different objectives and also the potentially different importance of multiple indicators in measuring achievement of the objectives needs to be developed. Finally, overall “scores” for each objective need to be derived taking into account these differences in importance. The methods associated with each stage of the process are detailed in the following sections.

### Identifying objectives and priorities

Clear objectives definition for any natural resource management is fundamental to management success [20]. Objectives provide a transparent guide to what the management aims to achieve, identify potential conflicting activities, guide elements of the decision making process, and ensure accountability of the management agency to the broader community [21]. Measures of the relative importance of different objectives are necessary in order to assess overall performance of management as well as determining which objectives require greater attention in terms of information collection and also potentially policy focus.

Numerous studies on assessing social and economic objectives and indicators have been undertaken for fisheries (e.g., [20, 22–24]) and coastal management (e.g., [25]). In many cases, objectives are not clearly defined, and a formal objective elicitation process is necessary. Fortunately for this study, GHHP had already defined a set of cultural, social and economic management objectives for the Harbour (Table 1) [16].

**Table 1:** Cultural, social and economic objectives.

Component	Objective
Cultural	Registered cultural heritage sites associated with the Harbour and waterways are protected
	The Gladstone community’s sense of identity and satisfaction with the condition of the Harbour is increased
Social	Maintain or improve easy access to the Harbour waters and foreshore for recreation and community uses
	Maintain or improve a safe Harbour for all users
	Enhance liveability and wellbeing in the region
Economic	The Gladstone Harbour is managed to support shipping, transport and a diversity of industries
	Economic activity in the Gladstone Harbour continues to generate social and economic benefits to the regional community
	Enhance values of recreational and environmental assets

A range of methods have been applied in the literature to assess objective weights, each with advantages and disadvantages [26–30]. In this study, we used a combination of simple ranking and direct ratings approaches. The Analytic Hierarchy Process (AHP) [31] was also tested. The direct rating measure, however, was found to have the lowest variance in an cases, suggesting a more consistent measure across the sample [14]. While the ranking approach has been previously used in coastal and natural resource management applications (e.g., [32–34]), its main role in this application was to provide a prompt for individuals, to allow them to determine their relative rankings before having to assign a measure to the strength of the ranking. Direct rating methods have been applied in a number of coastal and resource management studies [35–37].

Several studies have also suggested that the direct rating approach is the most reliable in test-retest studies [27, 38, 39]. For the direct rating approaches (the Max100 approach), the highest ranked sub-component is allocated 100 points and subsequent (lower) objectives are allocated less than 100 points [26]. The final weight is determined by:

$$w_{i,j}=S_{i,j}/\sum_{i}S_{i,j}$$ (1)

where *S*_*i*,*j*_ is the initial score given to each objective *i* (i.e. between 1 and 100) and *W*_*i*,*j*_ is the final weight used in the analysis. The objective weights were derived from data collected through an online survey of local residents (see section on data collection below).

### Indicators and measures

The range of potential key indicators associated with each objective was previously considered by the GHHP [16]. The appropriate measures associated with each indicator were derived through a project team workshop, and based on previous experiences in developing measures of social indicators for marine based industries [24, 40]. The key criteria for inclusion involved the measure:

1. Having a direct relationship with the indicator; and
2. Being measurable (either through survey or secondary data).

The set of candidate indicators and measures for each of the components of the report card were discussed with a focus group in the Gladstone region. This group represented a broad range of experience, with ages ranging from 18 to 65, years of experience living in the region ranging from 2 to 65 years, and an equal gender balance. Following the focus groups discussion, some additional measures were added and some others were removed or modified. Most measures remained unchanged. The final set of indicators and measures are given in Table 2.

**Table 2:** Key indicators and measures^a^.

Objective	Indicators	Measures
**Cultural Objective Indicators and Measures**		
Protection of cultural heritage sites	1. Perceptions that traditional sites are well protected	1. Community perception of level of traditional site protection
	2. Perception that traditional owners are appropriately consulted	2. Community perception of appropriate level of traditional owner consultation
	3. Condition of non-indigenous cultural heritage sites	3. Proportion of sites in good/average/poor condition (Secondary data)
	4. Proportion of known indigenous sites protected	4. Proportion of known sites protected (%, Secondary data)
Enhance sense of place	1. Distinctiveness/uniqueness of the area	1. Perceptions: No better place than here for my activities; This place is part of who I am
	2. Continuity	2. Proportion of life lived in area; Likely to stay at least 5 more years
	3. Self esteem	3. Proud to live in the area
	4. Self-efficacy	4. Perceptions: Harbour contributes to quality of life; Feel able to influence management
	5. Attitudes	5. Perceptions: Harbour is a) key part of the Gladstone community; b) great asset to region; c) great asset to Queensland
	6. Values	6. Perceptions of values including: variety of marine life; outdoor recreation opportunities; attraction of visitors to the region; enjoyment of scenery; spirituality; culture; historically significance
**Social Objective Indicators and Measures**		
Enhance access to the Harbour	1. Satisfaction with access to the Harbour	1. Satisfaction with harbour access
	2. Satisfaction with access to ramps and public spaces	2. Satisfaction with a) access to public spaces; b) number of ramps; c) frequency of use
	3. Perceptions of Harbour health	3. Perceptions of: a) condition of harbour; b) optimism about future harbour health; c) whether harbour health has improved over last 12 months
	4. Barriers to access	4. Perceptions of whether: a) marine debris is a problem; b) marine debris affects access; c) shipping reduces use; d) recreational boats reduce use
Enhance usability of the Harbour	1. Satisfaction with Harbour recreational activities	1. Satisfaction with: a) overall last trip; b) quality of ramps and facilities;
	2. Air and water quality	2. Satisfaction with: a) water quality; b) air quality. c) perception that water quality does not affect use
	3. Harbour safety	3. Perceptions: a) seafood is safe to eat; b) area is safe at night; Secondary data: c) relative occurrence of oil spills; d) relative occurrence of marine safety incidents
Enhance liveability and wellbeing in the region	1. Liveability measures	1. a) Perception: Harbour makes living in the area a better experience; b) actively participate in community events
**Economic Objective Indicators and Measures**		
Enhance economic performance of Harbour based industries	1. Commercial fishing performance	1. Relative performance of a) line fisheries; b) net fisheries; c) trawl fisheries; d) pot fisheries over time and similar regions (secondary data)
	2. Shipping activity	2. Relative level of shipping activity for both imports and exports over time (secondary data)
	3. Tourism	3. Relative value of tourism expenditure over time (secondary data)
Enhance economic stimulus to the broader community	1. Socio-economic status of the community	1. Relative standing of Index of Access to Economic Resources compared to other regions (secondary data and survey data)
	2. Employment	2. Relative standing of unemployment rate compared to other regions (secondary data)
Enhance values of recreational and environmental assets	1. Recreational non-market value	1. Satisfaction with a) beach recreation; b) recreational fishing; c) land based recreation; weighted by relative consumer surplus from travel cost analysis

^a)^ unless specified as secondary data, measures are derived from a community survey.

Many of the indicators for the social and cultural sub-components are difficult to quantify directly, as many are conceptual rather than tangible. As a result, several measures are associated with most indicators (effectively indicators of the indicators), and some associated with more than one indicator.

### Data collection

Most of the social and cultural measures are perceptions based, and were derived from a community survey. Information on these, and some economic measures, were collected using a computer assisted telephone interview (CATI) survey of residents in the Gladstone region. A telephone survey company was commissioned to obtain 400 responses, which were collected over a three week period. The survey questions were also trialled on the focus group mentioned in the previous section, and some further (minor) modifications to the survey questions were also undertaken after the first few interviews based on feedback from the survey company (i.e. questions that seemed to be difficult to understand). The interview guide for the CATI survey is presented in the supporting information **(**S1 File).

Most of the perception and satisfaction questions involved asking respondents their level of agreement on a 1–10 scale (where 1 = strongly disagree, 10 = strongly agree) with a series of statements related to each of the measures. Many of the statements were adjusted from previous research, particularly the Social and Economic Long Term Monitoring Program (SELTMP) for the Great Barrier Reef [41].

A subset of participants in the telephone survey was followed up with an on-line survey to elicit objective weightings. Individuals were asked to rank the different management objectives in order of their importance. Just over half (n = 218) telephone survey participants offered to participate in the on-line survey, and were emailed links to the survey. Of these, 83 actually completed the survey. A copy of the on-line survey is presented in the supporting information (S1 File). Full demographic details of the CATI and on-line survey participants are also provided in the supporting information (**Figs A and B and Tables A and B in** S2 File).

Similarly, a group of Australian social scientists with experience working in the coastal zone where surveyed, using an online survey instrument, to develop the links between the indicators and the objectives, and the measures to the indicators for the social and cultural models. Sixty social scientists were sent a link to the survey, from which 19 responded. A copy of the on-line survey is presented in the supporting information (S1 File).

Ethical clearance for the collection of the data, including the CATI survey, online surveys and focus group meetings, was approved by the CSIRO’s Human Research Ethics Committee (approval number: 072/14). The participants of the CATI survey were advised at the start of the interview about the confidential use of the data for research purposes and that consent to use the data for this purpose was collected verbally. Participation in the survey was voluntary, and withdrawal from the interview was possible at any time. Only completed interviews were used for the analysis, and only consenting participants were interviewed. Participants were also provided contact details for the project team if they wished to provide follow up comments about the interview. The survey was also promoted to the local community by the local media, including local newspapers and radio, prior to the telephone interviews commencing. Similarly, participants in the online surveys were provided with information sheets when sent the link to the survey, and were also further advised in the invitation email about the use of the data. Respondents were advised that participation in the online survey was voluntary and implied consent. Withdrawal through non-completion of the survey was also possible at any stage. In order to comply with ethical research standards, none of the participants are identifiable in this study. Verbal and implied consent was approved by the ethics committee given the nature of the data collection processes (i.e. data could not be obtained from non-consenting respondents).

Several of the social and cultural measures, and most of the economic measures, were derived from secondary data. These were also converted to a relative scale through either comparison over time (e.g., tourism expenditure), across regions (e.g., unemployment rate), or both (e.g., commercial fishing performance). For the fishing and shipping activities, a key measure used was capacity utilisation, which compares output at a particular point in time in the region to output in other similar regions and/or over time. Complete details on these analyses are presented in Pascoe et al [14]. The complete primary and secondary data collected and used in the study are available from the CSIRO Data Access Portal, http://doi.org/10.4225/08/567361B93A670.

### Development of a framework for assessing performance

The relationship between the measures, indicators and the objectives was developed using a Bayesian Belief Network (BBN) approach. Bayesian networks are essentially graphical models to which probabilities of certain outcomes given certain situations or observations can be assigned. These probabilities can be derived through the use of expert opinion, or derived from observations. Bayesian networks have been applied to coastal and marine resource management (particularly fisheries) in numerous cases, particularly when the effects of qualitative as well as quantitative factors are of interest (e.g., [24, 42–46]).

Bayesian network models provide a probability of an outcome rather than a discrete (deterministic) outcome. From the probability distribution, a mean (expected) outcome and confidence interval can be determined. How each indicator combines to inform the objective is determined through a combination of impact weighting and subjective assessment based on expert knowledge. The individual objective performances are aggregated to provide an overall component performance measure (i.e. economic, social, and cultural). A separate BBN was developed for each component.

BBN models are usually developed to model natural, social or economic systems, and to determine the probability of different outcomes occurring given the expected probability of different inputs and how these interact. Information on both observed inputs and outcomes is used to develop the models. In this case, we do not model a system *per se*, but use the BBN framework as a probabilistic means to aggregate different levels of information. The outcomes–the degree to which each objective is achieved–are unknown and undeterminable *a priori*, but instead are estimated using the framework given the observed inputs, and expert knowledge on how these inputs interact (through a series of conditional probability tables, or CPTs) to produce an expected but intangible outcome (i.e. report card grade). The models were developed in NETICA (www.norsys.com/netica.html).

**Fig 3:**
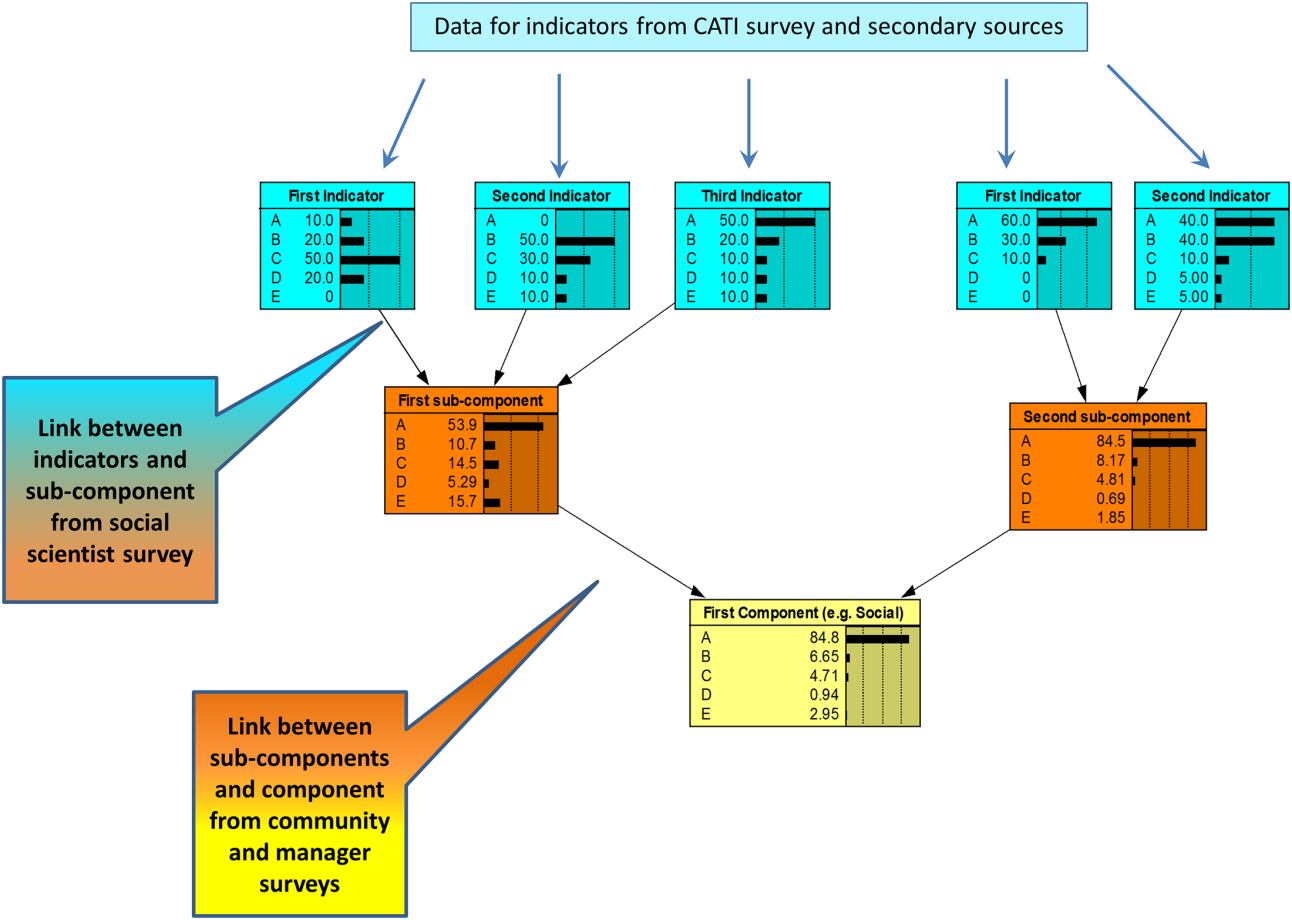
Data inputs into the BBN.

Inputs into the BBN were derived from different sources (Fig 3). The BBN was populated with data collected from the different surveys and other secondary sources in order to derive the preliminary report card scores. The information collected from the social science expert group was used to develop the conditional probability tables linking the measures to indicators and indicators to the social and cultural objectives. Similarly, the link between the objectives and main domain area (i.e. social, cultural and economic domains) were derived from the community online surveys. As heterogeneity is a common feature of objective weight elicitation, the distributions of the weights were incorporated directly into the CPTs, allowing uncertainty and heterogeneity to be simultaneously captured.

For the economic BBN, the weights derived from the community online survey were used to combine the three key objectives (see Table 1) and derive the CPTs. The R scripts used to estimate the CPTs are provided in the data files available from the CSIRO Data Access Portal, http://doi.org/10.4225/08/567361B93A670. Revenue shares were used to combine the results from the different industries in each part of the economic performance objective (e.g., different fishing fleets within the fishing activity, and also when combining fishing, tourism and other industries). For the recreational components, non-market values (consumer surplus estimates derived from a travel cost model analysis) were used to derive the weights, reflecting the relative importance of each recreational activity. For the final economic stimulus objective, equal weights were assigned to the indicators (see Table 2).

## Results

### Conditional probability tables (CPTs)

A key aim of the community and social scientist surveys was to develop conditional probability tables linking the indicator values to the objectives (social scientist survey) and the objectives to the overall component performance (community survey). Report cards generally use an A-E scoring system, with A being the best outcome and E being the worst. Generally, a C indicates an acceptable outcome, with D and E being unacceptable (i.e. a “fail”) [7]. Consistent with this approach, the distribution of results for each of the indicators was also presented as an A-E range. The aim of the CPTs is to derive a combined score of the child node given the scores of each contributing parent node.

To derive the CPTs, the potential combinations of outcomes (A-E) for each indicator were multiplied by the set of community preferences in order to determine the distribution of combined outcomes for the broader objective set

Formally, this is given by *O* = *WC*^*T*^, where *W* is an m*n matrix of weights, where *n* is the number of indicators, and *m* is the number of respondents, *C* is a *5*^n^*n matrix of objective outcome combinations. The product of these two matrices, *O*, is an m*5^*n*^ matrix representing the score individual *m* would give to the outcome (based on their individual weights) under the combination of outcomes (i.e. A,A; A,B; …, E,E). These are aggregated over all individuals to provide a probability of a particular objective outcome given a set of indicator outcomes. To operationalise the probability estimation, the outcomes A-E were given nominal values of [0.9,0.7,0.5,0.3,0.1], being the means of their ranges as defined by the surveys and other indicators.

The resultant probability of a particular outcome for the culture component given the individual objective outcomes are illustrated in Table 3. As expected, a combination of (A,A) produces an outcome of A for the main component with 100 percent probability. Conversely, a combination of (E,E) produces an outcome of E with 100 percent probability. Other combinations provide a mix of probabilities, reflecting the differences in the weightings of the different individual surveyed. For example, a combination of (A,B) results in a 12.5% probability of an A and an 87.5% probability of a B for the overall cultural component.

**Table 3:** Example of the conditional probability table results based on weights, cultural BBN.

Objectives		Cultural component outcome probability				
Heritage	Place	A	B	C	D	E
**A**	A	100.0	0.0	0.0	0.0	0.0
**A**	B	12.2	87.8	0.0	0.0	0.0
**A**	C	1.2	85.4	13.4	0.0	0.0
**A**	D	0.0	35.4	53.7	11.0	0.0
**A**	E	0.0	2.4	64.6	23.2	9.8
**B**	A	64.6	35.4	0.0	0.0	0.0
**B**	B	0.0	100.0	0.0	0.0	0.0
**B**	C	0.0	35.4	64.6	0.0	0.0
**B**	D	0.0	1.2	81.7	17.1	0.0
**B**	E	0.0	0.0	12.2	78.0	9.8
**C**	A	13.4	85.4	1.2	0.0	0.0
**C**	B	0.0	87.8	12.2	0.0	0.0
**C**	C	0.0	0.0	100.0	0.0	0.0
**C**	D	0.0	0.0	12.2	87.8	0.0
**C**	E	0.0	0.0	1.2	85.4	13.4
**D**	A	9.8	78.0	12.2	0.0	0.0
**D**	B	0.0	17.1	81.7	1.2	0.0
**D**	C	0.0	0.0	64.6	35.4	0.0
**D**	D	0.0	0.0	0.0	100.0	0.0
**D**	E	0.0	0.0	0.0	35.4	64.6
**E**	A	9.8	23.2	64.6	2.4	0.0
**E**	B	0.0	11.0	53.7	35.4	0.0
**E**	C	0.0	0.0	13.4	85.4	1.2
**E**	D	0.0	0.0	0.0	87.8	12.2
**E**	E	0.0	0.0	0.0	0.0	100.0

Similar tables were produced for the cultural and economic components, although as these are 125 rows long (i.e. *n*^*m*^ combinations where *n* is the number of grades (5) and *m* is the number of objectives (up to 3)) each are not presented below.

### Bayesian Belief Networks and report card scores

The data derived from the survey and other sources were applied to the three BBNs. These capture two forms of uncertainty–uncertainty in the indicator measure (through the distributions of results from the surveys) and uncertainty in indicator and objective importance (derived from the community and social science expert surveys).

**Fig 4:**
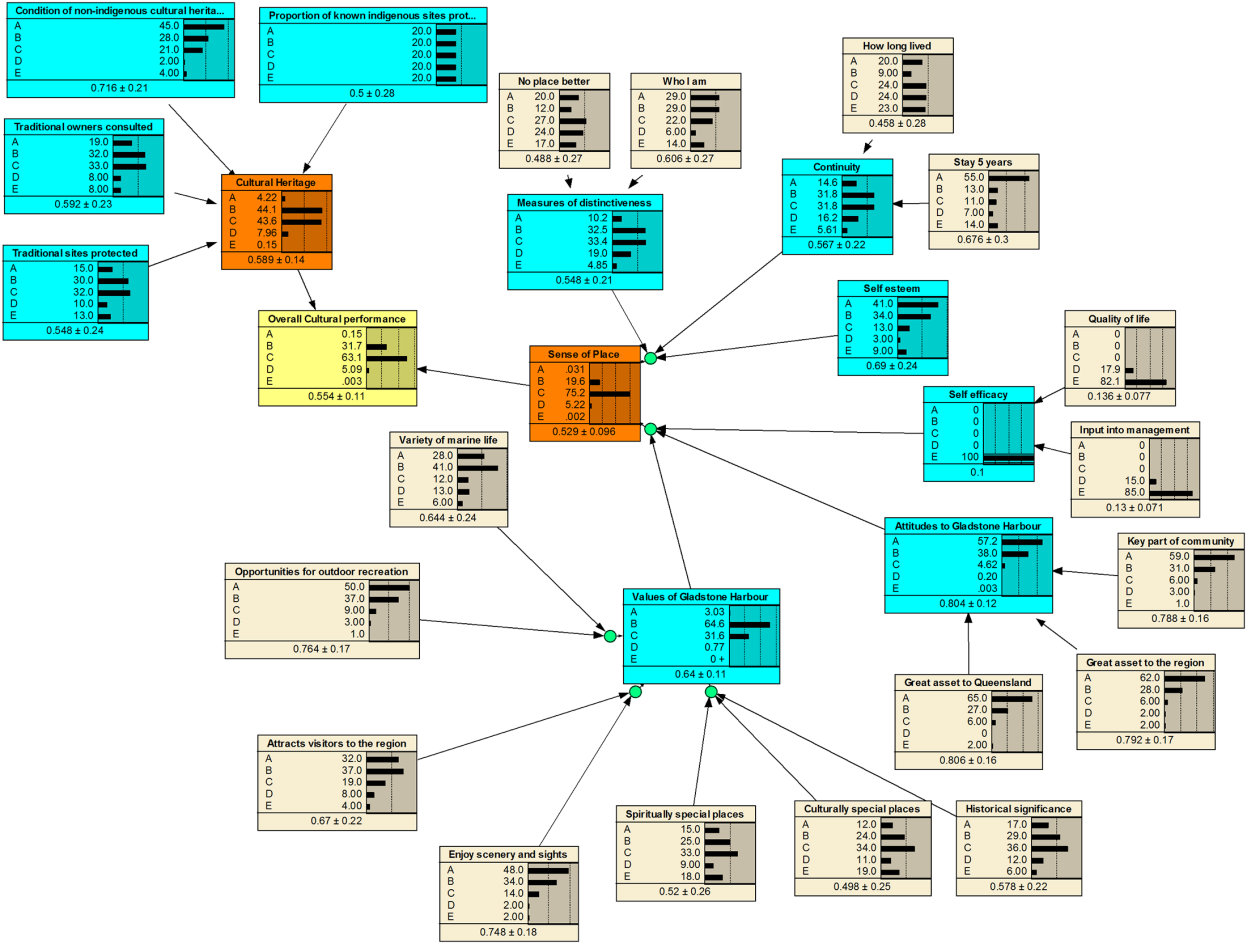
Fully populated cultural BBN with results.

**Fig 5:**
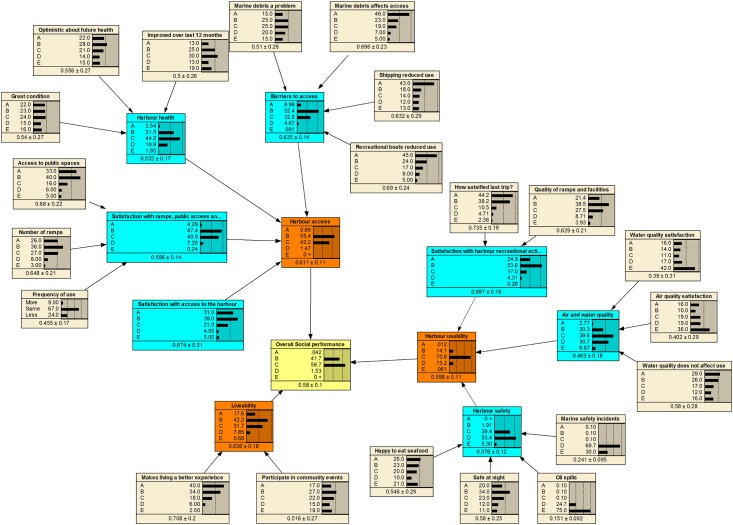
Fully populated social BBN with results.

**Fig 6:**
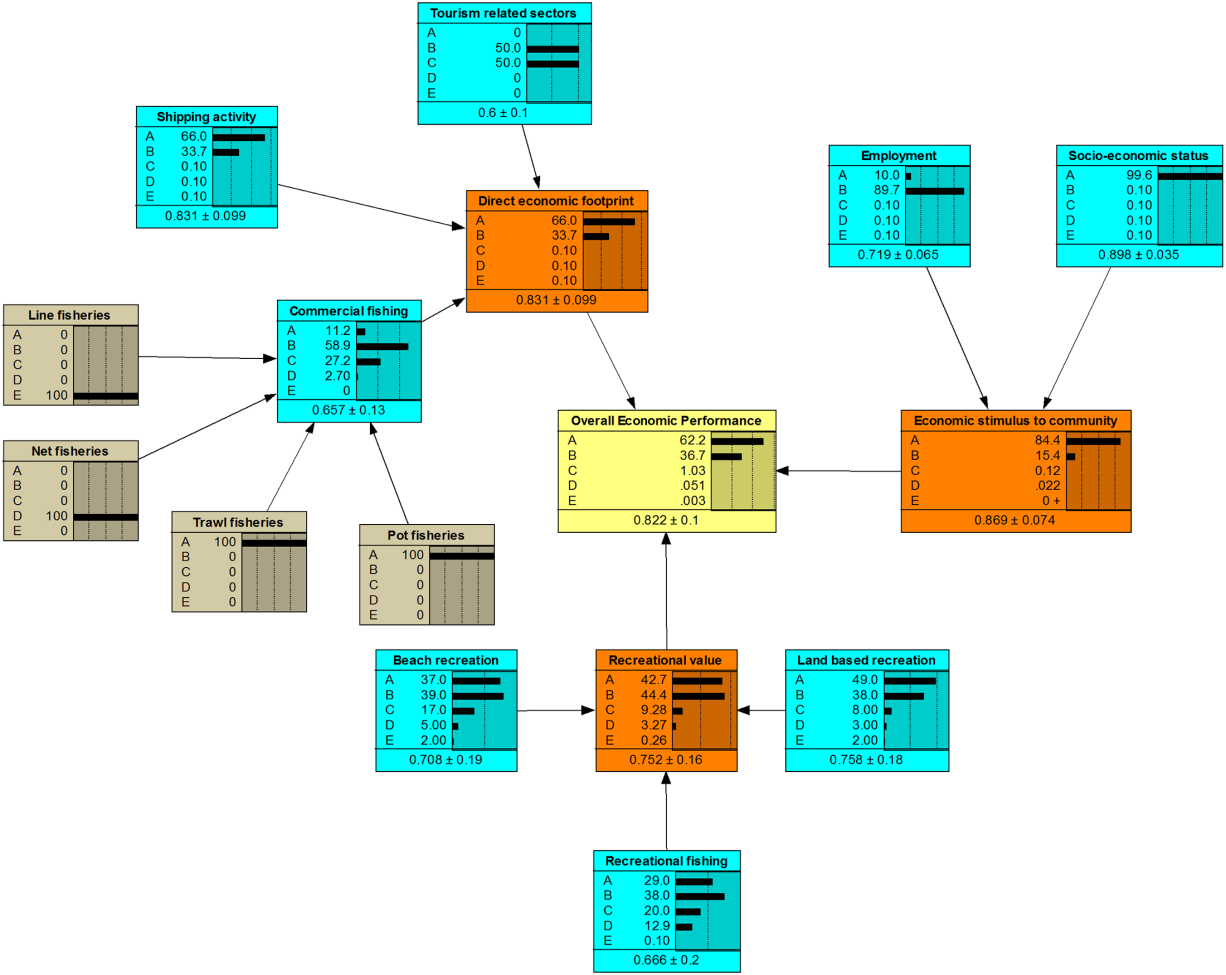
Fully populated economic BBN with results.

The fully populated BBNs are presented in Fig 4 (Cultural), Fig 5 (Social) and Fig 6 (Economic), based on the community priorities for each of the objectives. In these figures, the yellow box represents the final component outcome, the orange boxes the objectives, the blue boxes the indicators and the grey boxes the measures. The horizontal bars represent the probability of a particular outcome.

From Figs 4–6, the BBNs produce both a probability distribution of a given outcome based on the available information as well as a mean score (and standard deviation). In generating an average numeric score, each level has been allocated a score based on the midpoint of the potential range. For example, A has a range of 80–100%, so has a numeric score of 90%, C has a range of 40–60% with a numeric score of 50%, and E a range of 0–20% with a mid-point score of 10%. Subsequently, the BBN potentially produces a truncated range of numeric scores for each outcome of between 10% and 90%. The BBNs provide a graphical view of the uncertainty at each level of the score card, as well as the overall likelihood of different score card outcomes.

The results from the three BBNs are summarised in Table 4. While the scores are particular to the case study, they illustrate the information that can be derived from the BBNs. Report card scores are generally presented on an A-E scale, with a single score for each output. The average output from the BBN can also be converted to such a scale, based on the range of scores noted above.

**Table 4:** Report Card Scores derived from the BBNs.

	Weighted Mean Score	Std Deviation	Average BBN Grade
**Overall Cultural performance**	63%	11%	B
*Key indicators*			
•Attitudes to Gladstone Harbour	80%	12%	A
•Condition of non-indigenous cultural heritage sites	69%	23%	B
•Continuity	57%	22%	C
•Measures of distinctiveness	55%	21%	C
•Proportion of known indigenous sites protected	50%	28%	C
•Self-efficacy	55%	20%	C
•Self esteem	69%	24%	B
•Traditional owners consulted	59%	23%	C
•Traditional sites protected	55%	24%	C
•Values of Gladstone Harbour	64%	11%	B
*Objective scores*			
•Cultural Heritage	58%	14%	C
•Sense of Place	64%	10%	B
**Overall Social performance**	58%	10%	C
*Key indicators*			
•Air and water quality	46%	18%	C
•Barriers to access	64%	14%	B
•Harbour health	53%	17%	C
•Harbour safety	38%	12%	D
•Satisfaction with access to the Harbour	67%	21%	B
•Satisfaction with Harbour recreational activities	70%	16%	B
•Satisfaction with ramps, public access and spaces	60%	14%	C
*Objective scores*			
•Liveability	64%	18%	B
•Harbour usability	60%	11%	C
•Harbour access	61%	11%	B
**Overall Economic Performance**	82%	10%	A
*Key indicators*			
•Commercial fishing	66%	13%	B
•Shipping activity	83%	10%	A
•Tourism related sectors	60%	10%	B
•Land based recreation	76%	18%	B
•Beach recreation	71%	19%	B
•Recreational fishing	67%	20%	B
•Employment	72%	6%	B
•Socio-economic status	90%	3%	A
*Objective scores*			
•Direct economic footprint	83%	10%	A
•Economic stimulus to community	87%	7%	A
•Recreational value	75%	16%	B

From Table 4, management of the harbour could be considered to be performing relatively well, with most objectives receiving scores of between A and C. Overall, economic performance received an A grade, cultural performance a B grade and social performance a C grade. Potentially, an overall grade could be determined if weightings had been applied to the different components (i.e. social, cultural and economic) but this was not done at the request of the GHHP.

The BBNs can also be used to provide measures of the relative sensitivity of the outcomes to different measures and indicator values. Details on this are provided in the supporting information (**Tables C-E in** S2 File). From these, the relative “importance” of particular information can be determined, so that future data collection activities can be better focused.

## Discussion and Conclusions

Performance metrics are becoming increasingly common in many areas involving management of public assets and resources. In schools, universities and hospitals, for example, the development and publication of report cards are aimed at providing incentives for the individual institutions to improve their performance, as well as to identify which areas may be in need of increased attention. Similarly, report cards in environmental settings provide an incentive for improvement of resource and environmental management and feedback to managers on what is or is not working, and which areas may be in need of greatest change.

The key aim of this project was to develop and pilot a system for the collection and analysis of data relating to appropriate economic, social and cultural indicators for the purposes of developing a broader report card on the management of the Gladstone Harbour region. A socio-cultural-economic report card goes beyond what is normally considered in resource and environmentally focused report cards, which have tended to consider biophysical factors affected by the resource management. However, the move to management frameworks based around concepts of ecologically sustainable development (ESD) and ecosystem based management (EBM) requires consideration of the broader social and economic impacts and benefits of management as well as the environmental effects.

A key outcome of the project was a preliminary set of report card scores relating to each of the three components, demonstrating the feasibility of such an approach as a proof of concept. Fundamental to the development of a report card is the identification of a set of objectives against which performance is to be measured. These had largely been developed in previous studies, along with indicators to be assessed (and measures also) [16], so were not reconsidered in this study (although some additional measures and indicators were added following focus group discussions). The report card that was developed was based around these objectives, indicators and measures. In brief, they included measures of satisfaction with the Harbour, recreational amenities and the local environment, liveability measures, measures of cultural heritage protection and values relating to the cultural aspects of the Harbour, economic performance of Harbour based industries, recreational values and benefits to the local community.

Most of the information used in the analysis was derived from the community survey. While a fairly large community survey was required to collect this information, the information derived was directly applicable to the estimation of social and cultural performance measures. A key advantage of the survey information was that it was largely collected on a 1–10 scale, enabling the probability distributions of “satisfaction” to be directly incorporated into the BBN “models” of the region for each component. This also included a built-in benchmark, namely a score of fully satisfied (10), overcoming an issue experienced with other measures derived from secondary data (for which a benchmark needed to be determined).

The use of the BBN to tie together the different components and indicators in the report card worked well for several reasons. First, it allowed qualitative and highly subjective data to be combined with quantitative data in a consistent framework. Second, it allowed heterogeneity in the measures of the qualitative indicators (i.e. the community satisfaction information collected in the surveys) and differences in views of how important the different objectives were to be fully captured in the analysis. This heterogeneity is obfuscated by single aggregate metrics such as a mean value for each indicator or a single weight for each objective. Similarly, uncertainty in how the different indicators link to the objectives is also captured, rather than relying on a more deterministic model. Finally, the distribution of responses, measures and outcomes is depicted graphically at every level of the model. While a single metric is used to determine the final grade, the uncertainty around this is transparent, along with the uncertainty around each measurement and indicator value feeding into the final score.

Aggregation along the BBN requires additional information about the relative importance of a measure or indicator in determining the next level outcome. A number of different groups and approaches were used to develop the conditional probability tables that link the parent and child nodes in the BBN. Social science experts were surveyed to determine the key linkages between measures and indicators, and between indicators and sub-sub-components. Community members (a subset of those surveyed in the CATI survey) were surveyed to establish the relative importance of each objective, and to develop the links between these objectives and the overall component.

The use of experts for the technical components (linking measures to indicators to objectives) and community for the link between the objectives and components was found to be a useful approach. While there was heterogeneity in opinion about these relationships, this heterogeneity could be fully captured in the development of the conditional probability tables. This is a strength of the BBN approach, whereas such heterogeneity would be more problematic in more deterministic modelling or aggregation processes.

The use of expert opinion is a relatively common feature of BBNs, particularly when deriving the relationships between different nodes of the network. In our study, these relationships, and much of the data input, were derived from surveys of the general community as noted above. The use of community survey for populating the main measures and also the contingent probability tables in the BBN is a more democratic approach and avoids any potential bias associated with the selection and participation of different stakeholder groups

The approach developed for this study is broadly applicable to a wide range of coastal and other natural resource management situations. Considerable interest has already been expressed by other regions for a similar study. The approach allows the combination of largely qualitative social and cultural data to produce a quantitative metric of performance, which can also be considered in parallel with more quantitative economic and environmental performance measures. As such, it provides a useful approach for assessing coastal management performance under a broader triple bottom line and ecologically sustainable development framework.

## Supporting Information

S1 File Survey instruments used in the study. (PDF)

S2 File Demographic information of the survey sample and BBN sensitivity analysis. (PDF)
