# Supplementary material for: Developing a Social, Cultural and Economic Report Card for a Regional Industrial Harbour
Source: PLoS One. 2016 Feb 3;11(2):e0148271. doi: 10.1371/journal.pone.0148271 (PMC4740459; doi:10.1371/journal.pone.0148271)
Supplement: S1 File — (PDF) [file pone.0148271.s001.pdf]

Survey instruments used in the study

		Page
1.	CATI survey to collect social, cultural and economic indicator data	1.
2.	Community objective survey (online survey)	14.
3.	Managers objective survey (online survey)	21.
4.	Social scientist survey (online survey)	29.

GHHP social, cultural and economic indicators survey questions

To be read to respondents:

Hello! My name is ___________

We are calling you today to request your participation in a survey on the social and economic status of Gladstone Harbour. The project is funded by the Gladstone Healthy Harbours Partnership, and is being run by CSIRO, Central Queensland University and James Cook University. The aim of the project is to develop a report card on Gladstone harbour. We would like to ask you about your use of the Harbour and your perceptions about the harbour quality. The information will help managers to make better decisions about how the harbour is managed.

The survey will take about 15 mins to complete. Your participation is entirely voluntary and you are free to not answer any questions that you would prefer not to. All of your responses will remain strictly confidential.

Would you be happy to participate in this survey? Do you have any questions at this stage?

1) Do you live in the Gladstone region? Yes/No (screening question)
Possible age and gender screening questions here? -tba

2) In what suburb, town, or locality of the Gladstone region do you live? ____________

3) How long have you lived in the Gladstone region? ________ (years) ________ (months)

4) Do you own a boat? Yes/No

We will be asking you a number of questions about your use of Gladstone harbour and the surrounding areas. The area that we are interested in includes the coast and waters up to the Narrows, including Graham Creek, to the north, and extending south to Tannum Sands and Colosseum Bay. To the east it extends just past the east coast of Facing Island. We will call this the Gladstone Harbour area from now on.

5) When you think of the Gladstone Harbour area what are the first three words that come into your mind _______________ _________________ _________________ (exclude uninformative words e.g. the, it, like, well and plural words)

In this section of the survey we are going to ask you some questions about how you use the Gladstone Harbour area for recreation. We are going to ask you about three different types of recreational activity. The first relates to your use of beaches, the second to all water-based activity, and the third to shore-based activity.

6) a) In the previous 12 months, did you visit the Gladstone Harbour area at all? _______ Yes/No

   If yes: b) were any of these visits for recreation (not including visits where you paid a tour or ferry operator)? _________ Yes/No

7) In the previous 12 months, do you think you used the Gladstone harbour area for any recreation activity more or less often than the year before, or about the same? ______ More/ less / about the same

8) In the previous 12 months, how frequently did you use a boat ramp in the Gladstone Harbour area? *Please read out the list of categories (LHS).and record a single response in one of the two columns (some people might know the exact amount which is why we have provided the ranges) These instructions apply to all the frequency questions.*

**Response category**		**Range**	
**Never**		**0**	
4-7 times a week		150-300	
2-3 times a week		80-149	
About once a week		40-79	
About once every 2 weeks		20-39	
About once a month		7-19	
About 4-6 times a year		4-6	
3 times per year		3	
2 times per year		2	
About once a year		1	

9) In the previous 12 months have you visited the following beaches in the Gladstone Harbour area?

	Y/N
Barney Point	
Spinnaker Park artificial beach	
Boyne Is	
Tannum Sands	
Other (please specify)	

10) In the previous 12 months, how often have you visited a **beach** on the mainland in the Gladstone Harbour area? For example, Barney Point, Spinnaker Park artificial beach, Boyne Is, Tannum sands. Do not consider beaches further south than Tannum Sands.

**Response category**		**Range**	
**Never**		**0**	
4-7 times a week		150-300	
2-3 times a week		80-149	
About once a week		40-79	
About once every 2 weeks		20-39	
About once a month		7-19	
About 4-6 times a year		4-6	
3 times per year		3	
2 times per year		2	
About once a year		1	

We would now like you to think about any water-based activity you may have undertaken in the Gladstone harbour and surrounding area in the last year. We are interested in trips for boating, fishing (from a boat), water-sports, swimming etc.

We do not want you to include trips on the ferry or commercial boat cruises or other activities where you paid a commercial operator. We are also only interested in trips where you spend the majority of the trip in the Gladstone Harbour area. We are not interested in trips where you travelled through the harbour to get to somewhere else.

11) In the last 12 months, did you undertake any **boat-based recreational fishing** in the Gladstone Harbour? YES/NO
    a. If YES how often have you been **recreational fishing** in the Gladstone Harbour area?

**Response category**		**Range**	
**Never**		**0**	
4-7 times a week		150-300	
2-3 times a week		80-149	
About once a week		40-79	
About once every 2 weeks		20-39	
About once a month		7-19	
About 4-6 times a year		4-6	
3 times per year		3	
2 times per year		2	
About once a year		1	

12) In the last 12 months, did you undertake any of the following other water-based activities in the Gladstone Harbour area?

(Read the following list and get a yes/no response)

	Y/N
Motorised boating –general boat recreation	
Motorised water sports (e.g., waterskiing, jet-skiing)	
Non-motorised water sports (e.g. Kayaking, kite surfing, paddle boarding, rowing, windsurfing)	
Sailing	
Swimming	
Scuba or snorkelling	
Other(specify)	

a. IF YES In the last year, how often have you done other **water-based recreation** in the Gladstone Harbour area?

**Response category**		**Range**	
**Never**		**0**	
4-7 times a week		150-300	
2-3 times a week		80-149	
About once a week		40-79	
About once every 2 weeks		20-39	
About once a month		7-19	
About 4-6 times a year		4-6	
3 times per year		3	
2 times per year		2	
About once a year		1	

13) In the last 12 months did you undertake any of the following **shore-based activities** in the Gladstone Harbour area?
(Read the following list and get a yes/no response)

	Y/N
Walking	
Cycling	
Running	
Picnicking or barbecuing	
Shore-based fishing	
Relaxing by the water	
Sporting events	
Community events	
Other (specify)	

14) In the last year, how often have you done **shore-based recreation** in the Gladstone Harbour area?

Response category		Range	
**Never**		**0**	
4-7 times a week		150-300	
2-3 times a week		80-149	
About once a week		40-79	
About once every 2 weeks		20-39	
About once a month		7-19	
About 4-6 times a year		4-6	
3 times per year		3	
2 times per year		2	
About once a year		1	

15) We would now like you to think about the recreational activity or type of trip that you do **most frequently** in the Gladstone Harbour area**.**
Was this?

A beach recreation trip	
A recreational fishing trip	
Other water-based recreation (not fishing)	
A shore-based recreation trip	
Other, please specify	

16) Thinking back to the **last time** you did this activity (from the above question), how did you get to where you first accessed the Gladstone harbour area from your home? i.e. What form of transport did you use? (more than one response allowed)

Walk	
Bicycle	
Motor vehicle	
Other	

17) Approximately how many kilometres is it from your home to where you first accessed the harbour? ___________ kms

18) Approximately how long did it take to get there (one way) _________ hrs _____ mins

19) How many people did you go with? Count only those, including yourself, in the same vehicle as you.

No of adults (including yourself)	
No of children (16 yrs and under)	

20) Approximately how long did your recreational activity last? _________ hrs (use proportion if required)

21) Did you spend most of your time doing this activity or do other activities as well such as shopping or visiting friends?

Spent most of the time doing this activity	Yes/No
**If you did other things as well**, approximately what proportion of your time was spent doing the recreational activity<br>*Do not include travel time*	% of time

22) Did your activity involve the use of a boat or jet ski? __________ Yes/ No

If yes

23) Approximately how many kms or nautical miles did you travel by boat? __________ kms or __________________ nautical miles

24) Roughly how many Litres or $ worth of fuel did you use? __________L or $ _________

25) Thinking of this recreational trip to the Gladstone Harbour area, how satisfied were you overall with your experience? *On a scale for 1 to 10 where 1= very unsatisfied to 10= very satisfied.*

Very unsatisfied				Very slightly unsatisfied	Very slightly satisfied				Very satisfied
1	2	3	4	5	6	7	8	9	10
☐	☐	☐	☐	☐	☐	☐	☐	☐	☐

We are now going to ask you a few questions about the recreational facilities around the Gladstone harbour area.

Do you agree or disagree with the following statements on a scale from 1 to 10 with 1=strongly disagree to 10=strongly agree (*also allow a don't know or non response)*

	Strongly [illegible]				Very [illegible]	Very [illegible]				Strongly [illegible]	**No [illegible]**
	1	2	3	4	5	6	7	8	9	10	
26) I am satisfied with the level of access to public spaces around Gladstone Harbour											
27) I am satisfied with the number of boat ramps available in the Gladstone Harbour area											
28) I am satisfied with the quality of boat ramps, including associated facilities, available in the											

	Strongly Disagree				Very slightly	Very slightly				Strongly Agree	**No Answer**
Gladstone Harbour area											
29) I have fair access to Gladstone Harbour compared to other users of the harbour											
30) There are other places that are better than the Gladstone Harbour area for the recreational activities that I do											
31) The amount of shipping in Gladstone Harbour has reduced my use of the area											
32) The amount of recreational boating activity in Gladstone Harbour has reduced my use of the area											

We are now going to ask you some more general questions about your impression of the Gladstone harbour area.
Do you agree or disagree with the following statements on a scale from 1 to 10 with 1=strongly disagree to 10=strongly agree (*also allow a don't know or non response)*

With 1=strongly disagree to 10=strongly agree	Strongly Disagree				Very slightly	Very slightly				Strongly Agree	**No Answer**
	1	2	3	4	5	6	7	8	9	10	
33) The Gladstone Harbour area is not in great condition											
34) I feel optimistic about the future health of Gladstone Harbour											
35) The health of the harbour has improved in the past 12 months											
36) Marine debris and litter is a problem in Gladstone Harbour											
37) The amount of marine debris and litter in Gladstone Harbour affects my access to the area											
38)											
39)											

With 1=strongly disagree to 10=strongly agree	Strongly Disagree				Very slightly	Very slightly				Strongly Agree	**No Answer**

With 1=strongly disagree to 10=strongly agree	Strongly Disagree				Very slightly	Very slightly				Strongly Agree	**No Answer**
	1	2	3	4	5	6	7	8	9	10	
40) I am not concerned about water quality in the Gladstone Harbour area											
41) I am not concerned about air quality in the Gladstone Harbour area											
42) The water quality in Gladstone Harbour has not affected how often I use the area in the last 12 months											
43) I would be happy to eat seafood caught in the Gladstone Harbour area											
44) I feel safe being in the Gladstone Harbour area at night											
45) Gladstone Harbour makes living in Gladstone a better experience											
46) I regularly participate in community events in the Gladstone Harbour area											

We are now going to ask you some questions about your general perceptions on how the harbour is managed and how important it is to you.

Do you agree or disagree with the following statements (1-10)?

With 1=strongly disagree to 10=strongly agree	Strongly Disagree				Very slightly	Very slightly				Strongly Agree	**No Answer**
	1	2	3	4	5	6	7	8	9	10	
47) I feel able to have input into the management of the Gladstone Harbour if I choose to											
48) I believe the traditional sites and customs in the Gladstone Harbour area are well protected											
49) I believe the Traditional Owners of the Gladstone Harbour area are well consulted by the regional managers											

With 1=strongly disagree to 10=strongly agree	Strongly				Very	Very				Strongly	**No**
	1	2	3	4	5	6	7	8	9	10	
50) I feel proud that I live in the Gladstone community											
51) The Gladstone Harbour area is part of who I am											
52) The Gladstone Harbour area improves my quality of life											
53) I do not plan to be a resident of this region in the next 5 years											
54) The Gladstone Harbour is a key part of the Gladstone community											

We are now going to ask you questions about what you value about Gladstone harbour. Do you agree or disagree with the following statements (1-10)?

With 1=strongly disagree to 10=strongly agree	Disagree				Very	Very				Agree	**No**
	1	2	3	4	5	6	7	8	9	10	
55) I value the Gladstone Harbour area because it supports a variety of marine life											
56) I value the Gladstone Harbour area because it provides opportunities for outdoor recreation											
57) I value the Gladstone Harbour area because it attracts visitors to the region											
58) The Gladstone Harbour area is a great asset for the economy of this region											
59) The Gladstone Harbour area is a great asset for the economy of Queensland											
60) I value the Gladstone Harbour area because I enjoy the scenery and sights											
61) I value the Gladstone Harbour area because there are											

spiritually special places											
62) I value the Gladstone Harbour area because there are culturally special places											
63) I value the Gladstone Harbour area because it has historical significance that matters to me											

ECONOMIC AND DEMOGRAPHIC

We are now going to ask some questions about you and your household. This is to help us compare your responses with other studies in the area and also other respondents.

64) What is your age?

18-24	25-34	35-44	45-54	55-64	65+
☐	☐	☐	☐	☐	☐

65) Are you male ☐ or female? ☐

66) Do you identify as a traditional owner of the area? Yes ☐ No ☐

67) What is your approximate household income?

**Weekly**	**≤$399**	**$400 - $799**	**$800 - $1249**	**$1250 - $1499**	**$1500 - $1999**	**$2000 - $2499**	**$2500 - $2999**	**≥$3000**
**Annual**	**≤$20,799**	**$20,800-$41,599**	**$41,600-$64,999**	**$65,000-$77,999**	**$78,000-$103,999**	**$104,000-$129,999**	**$130,000-$155,999**	**≥$156,000**
	☐	☐	☐	☐	☐	☐	☐	☐

68) How many adults (> 18 years old) live in your household? ☐

69) How many children 15 years and older old live in your household? ☐

70) How many children younger than 15 years old live in your household? ☐

71) Is any adult in the household unemployed? (exclude stay at home mums/dads not actively seeking work, or retirees) Yes ☐ No ☐

72) Is any adult in the household self employed? Yes ☐ No ☐

73) Is your home:

Owned with a mortgage?	Owned without a mortgage?	Rented?
☐	☐	☐

a. I*f owned with a mortgage*, is your mortgage repayment greater than $3000/month
Yes ☐ No ☐

b. *If rented*, is your rent payment greater than $175/week
Yes ☐ No ☐

74) Does your household have a car? Yes ☐ No ☐

75) How many bedrooms does your house have? [ ]

Final questions: and then thank them for their participation

73. Would you be willing to complete a small exercise in another online survey. This is about determining the relative importance of the different indicators that will be using in the Gladstone Harbour report cord?
If yes, please collect an email address.____

74.This survey will be conducted on an annual basis to collect information for the Gladstone harbour report card. Would you be willing to be contacted again next year to answer some more questions about the Gladstone habour.
If yes, please collect an email address.____

That is the end of the survey

Combined results from the surveys will help ensure the opinions of the people living in the Gladstone area are considered in the management of the harbour. You will be able to access the final report online at the end of the year. If you wish to receive further information about the survey, I can give you the contact details for the project leader, Dr Sean Pascoe from the CSIRO, who can forward further details to you. Would you like these? (if yes then provide email sean.pascoe@csiro.au)

Thank you for your participation

GHHP Report Card
Objective importance survey

The aim of this survey is to determine community views on the relative importance of the stated objectives of the Gladstone Healthy Harbour Partnership (GHHP) (http://www.healthyharbour.org.au/). These views will help determine the degree to which the broader social, cultural and economic objectives of the Partnership are being achieved, and will be used in the development of a Report Card on the health of Gladstone Harbour.

The project to help develop the report card framework is being undertaken in partnership between the CSIRO Oceans and Atmosphere Flagship, Central Queensland University and James Cook University. You have already had some involvement with this project through the recent community telephone survey in which you particapted (thank you).

Why me?

You have been contacted as you have recently participated in a telephone survey collecting information for the development of the report card, and expressed a willingness to undertake a supplementary survey.

As an incentive to participate in the survey, we are offering the chance to win one of ten $50 gift vouchers. Your responses will be automatically linked to your email address, and ten respondents will be drawn at random on the **19th September 2014**.

Instructions

You will be presented with 3 different approaches to assessing the relative importance of the objectives.

1. The first is a simple ranking of objectives. You will be asked to rank the objectives from 1 to n (n=number of objectives).

2. The second is a scoring based system. You will be asked to allocate 100 points to the objective you have ranked number 1, then allocate points between 1-100 to the other objectives based on how important they are compared to the top ranked objective.

Both the first and second approaches involve choosing your response from a drop down menu.

3. In the third approach, you will be asked to assess the relative importance of sets of objectives. Two objectives will be presented at a time and you will be asked to indicate which you think is more important and by how much. If they are equally important you will be able to indicate that also.

What if I have problems completing the survey?

The survey should be relatively straight forward, and should take only 10 minutes or so to complete. If you do have problems please contact Sean Pascoe (email: sean.pascoe@csiro.au, phone: 07 3833 5966). There is also an opportunity to leave feedback or comments at the end of the survey.

Confidentiality
Your individual responses will not be identifiable in any form in any related output from the study. Only aggregate information will be released. The project team will have access to individual responses for the purposes of completing the analysis, but these will not be disclosed to any third party in an identifiable form.

Thank you for your further assistance with this project.

Cultural objectives

On this page, you will be asked to compare the two cultural objectives identified by the GHHP. These are:

*1. **Cultural heritage objective**: That registered cultural heritage sites associated with the harbour and waterways are protected*

*2. **Sense of place objective:** The Gladstone community's sense of identity and satisfaction with the condition of the harbour is increased. "Sense of place" is a combination of characteristics that makes a place special and unique. It involves the human experience in a landscape, the local knowledge and folklore. Areas that have a strong sense of place have an identity and character recognized immediately by a visitor and valued deeply by residents.*

***1. In the first column, please rank the general objective in order of importance to you using the drop down. You can rank objectives equally if they are equally important.**

In the second column, give the objective you scored highest (i.e. 1) in the first column a score of 100. Give each other objective an importance score relative to this using the drop down menu. For example, the second ranked objective may have score of 90 if they are very close in importance to you, or might have a score of 50 if you think the first ranked objective is twice as important as the second ranked objective. If you believe they are equaly important then both would have a score of 100.

	Rank (first or second)	Score (First=100)
Cultural heritage		
Sense of place		

***2. The next exercise asks you to compare the objectives directly against each other, and indicate which is the most important (and by how much). You can use the results from the first question as a guide. For example, if you scored objective A as 100 and objective B as 90, you might say that objective A is "*slightly more important*" than objective B, or even "*more important*", depending on how you feel about the strength of the relationship.**

- **Not all points are labelled. Unlabelled points are mid-way between the adjacent labelled points.**

	**A** is most important		**A** is much more important than **B**		**A** is more important than **B**		**A** is slightly more important than **B**	**Both are equally important**	**B** is slightly more important than **A**		**B** is more important than **A**		**B** is much more important than **A**		**B** is most important
(**A**) *Cultural heritage*; versus (**B**) *Sense of place*	○	○	○	○	○	○	○	○	○	○	○	○	○	○	○

Social objectives

On this page, you will be asked to compare the three social objectives identified by the GHHP

*1. **Harbour access**: Maintain/Improve easy access to the harbour waters and foreshore for recreation and community uses*
*2. **Harbour usability**: Maintain/Improve a safe harbour for all users (e.g. swimming, boating and foreshore activities)*
*3. **Liveability and wellbeing**: Contribution of the harbour to liveability of Gladstone*

***3. In the first column, please rank the general objective in order of importance to you using the drop down. You can rank objectives equally if they are equally important. In the second column, give the objective you scored highest (i.e. 1) in the first column a score of 100. Give each other objective an importance score relative to this using the drop down menu.**

	Rank (first, second, third)	Score (First=100)
Harbour access		
Harbour usability		
Liveability and wellbeing		

***4. The next exercise asks you to compare the objectives directly against each other, and indicate which is the most important (and by how much). You can use the results from the first question as a guide.**

- **Not all points are labelled. Unlabelled points are mid-way between the adjacent labelled points.**

	**A** is most important		**A** is much more important than **B**		**A** is more important than **B**		**A** is slightly more important than **B**	**Both are equally important**	**B** is slightly more important than **A**	**B** is more important than **A**		**B** is much more important than **A**		**B** is most important
(**A**) *Harbour access*; versus (**B**) *Harbour usability*	○	○	○	○	○	○	○	○	○	○	○	○	○	○	○
(**A**) *Harbour access*; versus (**B**) *Liveability and wellbeing*	○	○	○	○	○	○	○	○	○	○	○	○	○	○	○
(**A**) *Harbour usability*; versus (**B**) *Liveability and wellbeing*	○	○	○	○	○	○	○	○	○	○	○	○	○	○	○

Economic objectives

On this page, you will be asked to compare the three economic objectives identified by the GHHP

*1. **Economic performance of the Harbour industries:** The Gladstone Harbour is managed to support shipping, transport and a diversity of industries*
*2. **Economic stimulus to the community:** Economic activity in the Gladstone Harbour continues to generate social and economic benefits to the broader regional community*
*3. **Economic values of recreational and environmental assets**: Value of recreational activity in the Harbour and of the natural environmental assets is maintained or improved*

***5. In the first column, please rank the general objective in order of importance to you using the drop down. You can rank objectives equally if they are equally important. In the second column, give the objective you scored highest (i.e. 1) in the first column a score of 100. Give each other objective an importance score relative to this using the drop down menu.**

	Rank (first, second, third)	Score (First=100)
Economic performance of the Harbour industries		
Economic stimulus to the community		
Economic values of recreational and environmental assets		

***6. The next exercise asks you to compare the objectives directly against each other, and indicate which is the most important (and by how much). You can use the results from the first question as a guide.**

- **Not all points are labelled. Unlabelled points are mid-way between the adjacent labelled points.**

	**A** is most important		**A** is much more important than **B**		**A** is more important than **B**		**A** is slightly more important than **B**	**Both are equally important**	**B** is slightly more important than **A**	**B** is more important than **A**		**B** is much more important than **A**		**B** is most important	
(**A**) *Economic performance*; versus (**B**) *Economic stimulus to the community*	○	○	○	○	○	○	○	○	○	○	○	○	○	○	○
(**A**) *Economic performance*; versus (**B**) *Economic values of recreational and environmental assets*	○	○	○	○	○	○	○	○	○	○	○	○	○	○	○
(**A**) *Economic stimulus to the community*; versus (**B**) *Economic values of recreational and environmental assets*	○	○	○	○	○	○	○	○	○	○	○	○	○	○	○

Some questions about you and your answers

The purpose of these questions is to gain an idea of how confident you are about your responses, and how you found the survey questions.

*7. How confident are you about your responses relating to the different sub-objectives in each category?

	Very unsure	Unsure	Neither sure nor unsure	Sure	Very sure
Cultural objectives?	◯	◯	◯	◯	◯
Social objectives?	◯	◯	◯	◯	◯
Economic objectives?	◯	◯	◯	◯	◯

*8. How confident are you about your responses for each of the three approaches used?

	Very unsure	Unsure	Neither sure nor unsure	Sure	Very sure
Ranking (First, second, etc)	◯	◯	◯	◯	◯
Scoring (1-100)	◯	◯	◯	◯	◯
Direct comparisons (A vs B)	◯	◯	◯	◯	◯

Thank you

9. If you would like to be entered into the draw for the gift vouchers, please enter your details below:

Name

Email Address

10. Thank you for participating in this study. For further information about the survey and how it will be used, please contact Sean Pascoe (sean.pascoe@csiro.au). If you would like to make comments about the survey, please do so below.

GHHP Report Card
Objective importance survey

The aim of this survey is to determine key stakeholder, expert and community views on the relative importance of the stated objectives of the Gladstone Healthy Harbour Partnership (GHHP). These views will help determine the degree to which the broader social, cultural and economic objectives of the Partnership are being achieved, and will be used in the development of a Report Card on the health of Gladstone Harbour.

The project to help develop the report card framework is being undertaken in partnership between the CSIRO Oceans and Atmosphere Flagship, Central Queensland University and James Cook University.

Why me?

You have been contacted as you have a role either with the GHHP, with one of the partner organisations, or are involved with management of the harbour, region or with an industry affected by the Harbour to some degree.

Instructions

You will be presented with 3 different approaches to assessing the relative importance of the objectives.

1. The first is a simple ranking of objectives. You will be asked to rank the objectives from 1 to n (n=number of objectives).

2. The second is a scoring based system. You will be asked to allocate 100 points to the objective you have ranked number 1, then allocate points between 1-100 to the other objectives based on how important they are compared to the top ranked objective. For example, if the second ranked objective is close to the first, you may choose to allocate it 95 points. Conversely, if the second ranked objective is much less important, you may choose to allocate it 50 points. In either case, you would allocate the third ranked objective less points than the two higher ranked objectives depending on how important it was relative to the first objective. You may allocate equal points to some or all objectives if you feel they are equally important.For example, you may allocate both the second and third objective 80 points if both are highly important. With the exception of the first ranked objective (which has an allocation of 100 points always), you may allocate any number of points (less than 100) to the other objectives depending on how important you feel they are relative to the first objective.

Both the first and second approaches involve choosing your response from a drop down menu.

3. In the third approach, you will be asked to assess the relative importance of sets of objectives. Two objectives will be presented at a time and you will be asked to indicate which you think is more important and by how much. If they are equally important you will be able to indicate that also.

What if I have problems completing the survey?

The survey should be relatively straight forward, and should take only 10 minutes or so to complete. If you do have problems please contact Sean Pascoe (email: sean.pascoe@csiro.au, phone: 07 3833 5966). There is also an opportunity to leave feedback or comments at the end of the survey.

Confidentiality

Your individual responses will not be identifiable in any form in any related output from the study. Only aggregate information will be released. The project team will have access to individual responses for the purposes of completing the analysis, but these will not be disclosed to any third party in an identifiable form.

Thank you for your assistance with this project.

Cultural objectives

On this page, you will be asked to compare the two cultural objectives identified by the GHHP. These are:

*1. **Cultural heritage objective**: That registered cultural heritage sites associated with the harbour and waterways are protected*

*2. **Sense of place objective:** The Gladstone community's sense of identity and satisfaction with the condition of the harbour is increased.*
"Sense of place" is a combination of characteristics that makes a place special and unique. It involves the human experience in a landscape, the local knowledge and folklore. Areas that have a strong sense of place have an identity and character recognized immediately by a visitor and valued deeply by residents.

***1. In the first column, please rank the general objective in order of importance to you using the drop down. You can rank objectives equally if they are equally important.**

In the second column, give the objective you scored highest (i.e. 1) in the first column a score of 100. Give each other objective an importance score relative to this using the drop down menu. For example, the second ranked objective may have score of 90 if they are very close in importance to you, or might have a score of 50 if you think the first ranked objective is twice as important as the second ranked objective. If you believe they are equaly important then both would have a score of 100.

	Rank (first or second)	Score (1-100)
Cultural heritage		
Sense of place		

***2. The next exercise asks you to compare the objectives directly against each other, and indicate which is the most important (and by how much). You can use the results from the first question as a guide. For example, if you scored objective A as 100 and objective B as 90, you might say that objective A is "*slightly more important*" than objective B, or even "*more important*", depending on how you feel about the strength of the relationship.**

- **Not all points are labelled. Unlabelled points are mid-way between the adjacent labelled points.**

	**A** is most important		**A** is much more important than **B**		**A** is more important than **B**		**A** is slightly more important than **B**	**Both are equally important**	**B** is slightly more important than **A**		**B** is more important than **A**		**B** is much more important than **A**		**B** is most important
(**A**) *Cultural heritage*; versus (**B**) *Sense of place*	○	○	○	○	○	○	○	○	○	○	○	○	○	○	○

Social objectives

On this page, you will be asked to compare the three social objectives identified by the GHHP

*1. **Harbour access**: Maintain/Improve easy access to the harbour waters and foreshore for recreation and community uses*
*2. **Harbour usability**: Maintain/Improve a safe harbour for all users (e.g. swimming, boating and foreshore activities)*
*3. **Liveability and wellbeing**: Contribution of the harbour to liveability of Gladstone*

***3. In the first column, please rank the general objective in order of importance to you using the drop down. You can rank objectives equally if they are equally important.**

In the second column, give the objective you scored highest (i.e. 1) in the first column a score of 100. Give each other objective an importance score relative to this using the drop down menu. For example, the second ranked objective may have score of 90 if they are very close in importance to you, or might have a score of 50 if you think the first ranked objective is twice as important as the second ranked objective. If you believe they are equaly important then both would have a score of 100. The scores for the third will be less than (or equal to) the score of the second objective

	Rank (first, second, third)	Score (1-100)
Harbour access		
Harbour usability		
Liveability and wellbeing		

***4. The next exercise asks you to compare the objectives directly against each other, and indicate which is the most important (and by how much). You can use the results from the first question as a guide. For example, if you scored objective A as 100 and objective B as 90, you might say that objective A is "*slightly more important*" than objective B, or even "*more important*", depending on how you feel about the strength of the relationship.**

- **Not all points are labelled. Unlabelled points are mid-way between the adjacent labelled points.**

	**A** is most important		**A** is much more important than **B**		**A** is more important than **B**		**A** is slightly more important than **B**	**Both are equally important**	**B** is slightly more important than **A**		**B** is more important than **A**		**B** is much more important than **A**		**B** is most important
(**A**) *Harbour access*; versus (**B**) *Harbour usability*	○	○	○	○	○	○	○	○	○	○	○	○	○	○	○
(**A**) *Harbour access*; versus (**B**) *Liveability and wellbeing*	○	○	○	○	○	○	○	○	○	○	○	○	○	○	○
(**A**) *Harbour usability*; versus (**B**) *Liveability and wellbeing*	○	○	○	○	○	○	○	○	○	○	○	○	○	○	○

Economic objectives

On this page, you will be asked to compare the three economic objectives identified by the GHHP

*1. **Economic performance of the Harbour industries:** The Gladstone Harbour is managed to support shipping, transport and a diversity of industries*
*2. **Economic stimulus to the community:** Economic activity in the Gladstone Harbour continues to generate social and economic benefits to the broader regional community*
*3. **Economic values of recreational and environmental assets**: Value of recreational activity in the Harbour and of the natural environmental assets is maintained or improved*

***5. In the first column, please rank the general objective in order of importance to you using the drop down. You can rank objectives equally if they are equally important.**

In the second column, give the objective you scored highest (i.e. 1) in the first column a score of 100. Give each other objective an importance score relative to this using the drop down menu. For example, the second ranked objective may have score of 90 if they are very close in importance to you, or might have a score of 50 if you think the first ranked objective is twice as important as the second ranked objective. If you believe they are equaly important then both would have a score of 100. The scores for the third will be less than (or equal to) the score of the second objective

	Rank (first, second, third)	Score (1-100)
Economic performance of the Harbour industries		
Economic stimulus to the community		
Economic values of recreational and environmental assets		

***6. The next exercise asks you to compare the objectives directly against each other, and indicate which is the most important (and by how much). You can use the results from the first question as a guide. For example, if you scored objective A as 100 and objective B as 90, you might say that objective A is "*somewhat more important*" than objective B, or even "*more important*", depending on how you feel about the strength of the relationship.**

- **Not all points are labelled. Unlabelled points are mid-way between the adjacent labelled points.**

	**A** is most important		**A** is much more important than **B**		**A** is more important than **B**		**A** is slightly more important than **B**	**Both are equally important**	**B** is slightly more important than **A**		**B** is more important than **A**		**B** is much more important than **A**		**B** is most important
(**A**) *Economic performance*; versus (**B**) *Economic stimulus to the community*	○	○	○	○	○	○	○	○	○	○	○	○	○	○	○
(**A**) *Economic performance*; versus (**B**) *Economic values of recreational and environmental assets*	○	○	○	○	○	○	○	○	○	○	○	○	○	○	○
(**A**) *Economic stimulus to the community*; versus (**B**) *Economic values of recreational and environmental assets*	○	○	○	○	○	○	○	○	○	○	○	○	○	○	○

Some questions about you and your answers

The purpose of these questions is to gain an idea of how confident you are about your responses, which groups you think you represent (may be more than one); and how you found the survey questions.

*7. Which group(s) do you think you represent? You may tick more than one

- [ ] Harbour based industry
- [ ] Harbour management
- [ ] Environmental/conservation management
- [ ] Tourism industry
- [ ] Fishing (recreational and commerical)
- [ ] Other local industry
- [ ] Local community
- [ ] Traditional owners
- [ ] Scientific/expert advice
- [ ] Other (please specify)

*8. How confident are you about your responses relating to the different sub-objectives in each category?

	Very unsure	Unsure	Neither sure nor unsure	Sure	Very sure
Cultural objectives?	◯	◯	◯	◯	◯
Social objectives?	◯	◯	◯	◯	◯
Economic objectives?	◯	◯	◯	◯	◯

*9. How confident are you about your responses for each of the three approaches used?

	Very unsure	Unsure	Neither sure nor unsure	Sure	Very sure
Ranking (First, second, third)	◯	◯	◯	◯	◯
Scoring (1-100)	◯	◯	◯	◯	◯
Pairwise comparisons (A vs B)	◯	◯	◯	◯	◯

Thank you

10. If you would like us to contact you for any reason about the survey, please leave your contact details below.

**Name**	
**Company**	
**Email Address**	
**Phone Number**	

11. Thank you for participating in this study. For further information about the survey and how it will be used, please contact Sean Pascoe (sean.pascoe@csiro.au). If you would like to make comments about the survey, please do so below.

Mapping social and cultural indicators to objectives

The Gladstone Health Harbour Partnership (GHHP) have commissioned CSIRO, JCU and CQU to look to developing a social, cultural and economic "report card" on the health of the Harbour. A separate community survey is underway collecting information on a range of indicators that will be used in the development of the annual report card. As part of the study, we need to link the indicators to the set of objectives defined by the GHHP. The aim of this is to develop a measure of how well the objective has been achieved based on the set of indicators.

The aim of this survey is to determine key expert views on the relative importance of the different indicators in terms of determining the outcomes of the stated objectives of the GHHP, and also how much weight to give to each question response when measuring the indicators (where more than one question relates to an indicator). These views will help determine the degree to which the broader social and cultural objectives of the Partnership are being achieved, and will be used in the development of a Report Card on the social and cultural health of Gladstone Harbour.

Why me?

You have been contacted as you a recognised expert in terms of social and/or cultural aspects of marine and coastal management.

Participation in the survey is voluntary, but as there are relatively few experts like yourself in Australia we are hoping you will agree to participate. We are tracking who responds to the survey mainly so that we can send a reminder to non-respondents. As an incentive to participate, we are offering **two prizes of a $75 gift card**. We will randomly draw the two winners from the list of those who have completed the survey by the **19th September 2014.**

Instructions

You will be presented with 2 different approaches to assessing the relative relevance of the indicators in relation to the objectives. Both approaches involve choosing your response from a drop down menu.

1. The first is a simple ranking of indicator relevance. You will be asked to rank the indicators from first to nth (n=number of indicators).

2. The second is a scoring based system. You will be asked to allocate 100 points to the indicator you have ranked number 1, then allocate points between 1-100 to the other indicators based on how relevant they are compared to the top ranked indicator. (Unfortunately, SurveyMonkey is not able to automatically allocate 100 points to the first-ranked indicator, so you will have to do this manually - sorry).

What if I have problems completing the survey?

The survey should be relatively straight forward, although some of the comparisons might be tricky. Please do your best in terms of assigning a score to each indicator. Equal scores and ranks are OK if you truely believe that they have equal relevance. The survey should take between 15-20 minutes to complete (probably closer to 20). If you do have problems please contact Sean Pascoe (email: sean.pascoe@csiro.au, phone: 07 3833 5966). There is also an opportunity to leave feedback or comments at the end of the survey.

Confidentiality

Your individual responses will not be identifiable in any form in any related output from the study. Only aggregate information will be released. The project team will have access to individual responses for the purposes of completing the analysis, but these will not be disclosed to any third party in an identifiable form.

Thank you for your assistance with this project.

Cultural heritage objective and indicators

The first set of indicators relates to the Cultural heritage objective of the GHHP:

*1. **Cultural heritage objective**: That registered cultural heritage sites associated with the harbour and waterways are protected*

This objective considers both indigenous and non-indigenous cultural heritage, and is based on four indicators - two based on perceptions and the others based on measures:

1. A perception based indicator from the community survey asking "*I believe the traditional sites and customs in the Gladstone Harbour area are well protected*" with a 10 point scale (1= strongly disagree; 10=strongly agree);
2. A perception based indicator from the community survey asking "*I am confident the Traditional Owners of the Gladstone Harbour region are well considered by management*" with a 10 point scale (1= strongly disagree; 10=strongly agree);
3. A measure of the condition of registered non-indigenous cultural heritage sites; and
4. The proportion of known indigenous cultural heritage sites protected.

In the first column, please rank the general indicator from first to fourth in order of relevance in describing how well the objective is met. You can rank indicators equally if they are equally important (e.g. first, second, third, third).

In the second column, give the indicator you scored highest (i.e. first) in the first column a score of 100. Give each other indicator an importance score relative to this using the drop down menu. For example, a lower ranked indicator may have score of 90 if you believe it is very close in relevance, or might have a score of 35 if you think it is much less relevant than the first ranked indicator. If you believe indicators are equally relevant then both would have an equal score (e,g. using the above example you may score them 100,90,35,35).

*Cultural heritage indicators

	Rank (first to fourth)	Score (set First=100)
Perception: "*I believe the traditional sites and customs in the Gladstone Harbour area are well protected*"		
Perception: "*I believe the Traditional Owners of the Gladstone Harbour region are well consulted by regional managers*"		
Measure: Condition of non-indigenous cultural heritage sites		
Measure: Proportion of known indigenous sites protected		

Sense of place objective and indicators

The second set of indicators relates to the "Sense of place" objective of the GHHP:

Sense of place objective: *The Gladstone community's sense of identity and satisfaction with the condition of the harbour is increased.*

"Sense of place" is a combination of characteristics that makes a place special and unique. It involves the human experience in a landscape, the local knowledge and folklore. Areas that have a strong sense of place have an identity and character recognized immediately by a visitor and valued deeply by residents.

The indicators are all derived from a community survey and relate to perceptions of the local residents. There are two levels of indicator. The first level is the broad indicator and the second level consists of the questions relating to the broad indicator (each using a 10 point scale (1= strongly disagree; 10=strongly agree)). Several questions may relate to each indicator. The broad indicators and related survey questions are:

1) Measures of distinctiveness/uniqueness

- *There are no other places that are better than the Gladstone Harbour region for the recreational activities that I do*
- *The Gladstone harbour region is part of who I am*

2) Continuity

- *How long have you lived in the Gladstone region?*
- *I plan to be a resident of this region in the next 5 years*

3) Self esteem

- *I feel proud that I live in the Gladstone community*

4) Self efficacy

- *The Gladstone Harbour region improves my quality of life*
- *I feel able to have input into the management of the Gladstone Harbour if I choose to*

5) Attitudes to Gladstone Harbour

- *The Gladstone Harbour is a key part of the Gladstone community*
- *The Gladstone Harbour region is a great asset for the economy of this region*
- *The Gladstone Harbour region is a great asset for the economy of Queensland*

6) Values of Gladstone Harbour

- *I value the Gladstone Harbour region because it supports a variety of marine life*
- *I value the Gladstone Harbour region because it provides opportunities for outdoor recreation*
- *I value the Gladstone Harbour region because it attracts visitors to the region*
- *I value the Gladstone Harbour region because I enjoy the scenery and sights*
- *I value the Gladstone Harbour region because there are spiritually special places*
- *I value the Gladstone Harbour region because there are culturally special places*
- *I value the Gladstone Harbour region because it has historical significance that matters to me*

In the first column, please rank the general indicator in order of importance/relevance in describing how the "Sense of place" objective is met. You can rank indicators equally if they are equally relevant. For example, you may believe that distinctiveness is the main factor affecting sense of place, and continuity the least. In this case you would rank distinctivess first and continuity last. You will be given an opportunity to assess how the actual questions relate to the indicators in the next question.

In the second column, give the indicator you ranked first in the first column a score of 100. Give each other indicator a score relative to this using the drop down menu.

*Broad indicators of "Sense of place"

	Rank (first to sixth)	Score (First=100)
Measures of distinctiveness/uniqueness		
Measures of continuity		
Measures of self esteem		
Measures of self efficacy		
Attitudes to Gladstone Harbour		
Values of Gladstone Harbour		

Sense of place: linking survey questions to indicators

Most of the survey key indicators of "sense of place" have several underlying questions. The aim of this part of the survey is to determine how to asses the overall indicator given the range of different responses to each question by assigning weights to each question. You may believe they are all equal, or that responses to some questions are more influential than others.

As before, in the first column, please rank the survey question in order of relevance in describing the indicator. You can rank questions equally if they are equally relevant.

In the second column, give the question you scored first in the first column a score of 100. Give each other question a score relative to this using the drop down menu based on its relevance to the indicator. You can give equal scores to the questions if you think they equally relate to the indicator.

* Measures of distinctiveness/uniqueness

	Rank (first, second)	Score (First=100)
"There are no other places that are better than the Gladstone Harbour region for the recreational activities that I do"		
"The Gladstone harbour area is part of who I am"		

* Continuity

	Rank (first, second)	Score (First=100)
Length of time living in the Gladstone region		
"I plan to still be a resident of this region in the next five years"		

* Self efficacy

	Rank (first, second)	Score (First=100)
"*The Gladstone Harbour area improves my quality of life*"		
"I feel able to have input into the management of the Gladstone Harbour if I choose to"		

Sense of place: linking survey questions to indicators (continued)

As before, in the first column, please rank the survey question in order of relevance in describing the indicator. You can rank questions equally if they are equally relevant. In the second column, give the question you scored first in the first column a score of 100. Give each other question a score relative to this using the drop down menu based on its relevance to the indicator. You can give equal scores to the questions if you think they equally relate to the indicator.

*Attitudes to Gladstone Harbour

	Rank (first, second, third)	Score (First=100)
"The Gladstone Harbour is a key part of the Gladstone community"		
"The Gladstone Harbour area is a great asset for the economy of this region"		
"The Gladstone Harbour area is a great asset for the economy of Queensland"		

*Values of Gladstone Harbour

	Rank (first to seventh)	Score (First=100)
"I value the Gladstone Harbour area because it supports a variety of marine life"		
"I value the Gladstone Harbour area because it provides opportunities for outdoor recreation"		
"I value the Gladstone Harbour area because it attracts visitors to the region"		
"I value the Gladstone Harbour area because I enjoy the scenery and sights"		
"I value the Gladstone Harbour area because there are spiritually special places"		
"I value the Gladstone Harbour area because there are culturally special places"		
"I value the Gladstone Harbour area because it has historical significance that matters to me"		

Harbour access objective

The next set of indicators relates to the Harbour access objective of the GHHP:

***Harbour access**: Maintain or improve easy access to the harbour waters and foreshore for recreation and community uses*

The objective has four main indicators, each which involve one or more questions:

1) Satisfaction with access to the harbour

- *I have fair access to Gladstone Harbour compared to other users of the harbour*

2) Satisfaction with availability of ramps, public access and spaces

- *I am satisfied with the level of public access and spaces to the Gladstone Harbour area*
- *I am satisfied with the number of boat ramps available in the Gladstone Harbour area*
- *Do you think you used the harbour and surrounding area for any recreation activity mentioned above more or less often than the year before, or about the same? (More/ less / about the same)*

3) Perceptions on health of the harbour

- *The Gladstone Harbour area is in great condition*
- *I feel optimistic about the future health of the Gladstone Harbour*
- *The health of the harbour has improved in the last 12 months*

4) Barriers to access and use of the harbour

- *Marine debris and litter is a problem in Gladstone Harbour*
- *The amount of marine debris and litter in Gladstone Harbour affects my access to the area*
- *The amount of shipping activity in Gladstone Harbour has reduced my use of the area*
- *The amount of recreational boating activity in Gladstone Harbour has reduced my use of the area*

Information is also collected in the community survey about frequency of use of the harbour, activities undertaken and boat ownership. These information will be used for contextualising the report card outcomes rather than as an input into the report card per se.

The aim of this part of the survey is to determine the relative importance of each broader indicator in defining "Harbour access", based on the description of each indicator (in terms of the underlying questions). You may believe all are equally important or that some elements are more important than others.

In the first column, please rank the general indicator in order of relevance in describing how the "Harbour access" objective is met given the context of the questions used in measuring the indicator (above). You can rank indicators equally if they are equally relevant. In the second column, give the indicator you ranked first in the first column a score of 100. Give each other indicator a score relative to this using the drop down menu.

*Main indicators relating to the harbour access objective

	Rank (first to fourth)	Score (First=100)
Satisfaction with access to the harbour		
Satisfaction with availability of ramps, public access and spaces		
Perceptions of health of the harbour		
Barriers to access		

Harbour access objective: linking survey questions to the indicators

Most of the survey key indicators of "harbour access" have several underlying questions. The aim of this part of the survey is to determine how to asses the overall indicator given the range of different responses to each question by assigning weights to each question. You may believe they are all equal, or that responses to some questions are more influential than others.

As before, in the first column, please rank the general indicator in order of relevance in describing how the "Harbour access" objective is met given the context of the questions used in measuring the indicator (above). You can rank indicators equally if they are equally relevant. In the second column, give the indicator you ranked first in the first column a score of 100. Give each other indicator a score relative to this using the drop down menu.

*Satisfaction with availability of ramps, public access and spaces

	Rank (first to third)	Score (First=100)
"I am satisfied with the level of access to public spaces around Gladstone Harbour"		
"I am satisfied with the number of boat ramps available in the Gladstone Harbour area"		
Change in Usage: *"Do you think you used the harbour and surrounding area for any recreation activity more or less often than the year before, or about the same?"*		

*Perceptions of health of the harbour

	Rank (first to third)	Score (First=100)
"The Gladstone Harbour region is in great condition"		
"I feel optimistic about the future health of the Gladstone Harbour"		
"The health of the harbour has improved in the last 12 months?"		

*Barriers to access

	Rank (first to fourth)	Score (First=100)
"Marine debris and litter is a problem in Gladstone Harbour"		
"The amount of marine debris and litter in Gladstone Harbour affects my access to the area"		
"The amount of shipping in Gladstone Harbour has reduced my use of the area"		
"*The amount of recreational boating activity in Gladstone Harbour has reduced my use of the area*"		

Harbour usability objective

The next set of indicators relates to the Harbour usability objective of the GHHP:

***Harbour usability**: Maintain or improve a safe harbour for all users (e.g. swimming, boating and foreshore activities)*

The objective has three main indicators, each which involve one or more questions:

1) Satisfaction with harbor recreational activities

- *Thinking of your most recent recreational trip to the Gladstone Harbour region, how satisfied were you overall with your experience?*
- *I am satisfied with the quality of boat ramps, including associated facilities, available in the Gladstone Harbour area*

2) Perceptions of air and water quality in the harbour area

- *I am not concerned about water quality in the Gladstone Harbour area*
- *I am not concerned about air quality in the Gladstone Harbour area*
- *The water quality in Gladstone Harbour has not affected how often I use the area in the last 12 months*

3) Safety of harbour for public usage

- *I would be happy to eat seafood caught in the Gladstone Harbour area*
- *I feel safe being in the Gladstone Harbour area at night*
- Measure: Number of oil and chemical spills in the Harbour
- Measure: Number of marine safety incidents in the Harbour

The aim of this part of the survey is to determine the relative importance of each broader indicator in defining "Harbour usability", based on the description of each indicator (in terms of the underlying questions). You may believe all are equally important or that some elements are more important than others.

In the first column, please rank the general indicator in order of relevance in describing how the "Harbour access" objective is met given the context of the questions used in measuring the indicator (above). You can rank indicators equally if they are equally relevant. In the second column, give the indicator you ranked first in the first column a score of 100. Give each other indicator a score relative to this using the drop down menu.

*Main indicators relating to the harbour usability objective

	Rank (first to third)	Score (First=100)
Satisfaction with harbour recreational activities		
Perceptions of water quality		
Safety of harbour for public usage		

Harbour usability objective: linking survey questions to the indicators

Most of the survey key indicators of "harbour usability" have several underlying questions. The aim of this part of the survey is to determine how to asses the overall indicator given the range of different responses to each question by assigning weights to each question. You may believe they are all equal, or that responses to some questions are more influential than others.

* Satisfaction with harbour recreational activities

	Rank (first to second)	Score (First=100)
"Thinking of your most recent recreational trip to the Gladstone Harbour region, how satisfied were you overall with your experience?"		
"I am satisfied with the quality of boat ramps available in the Gladstone Harbour area"		

* Perceptions of air and water quality

	Rank (first to third)	Score (First=100)
"I am not concerned about water quality in the Gladstone Harbour area"		
"I am not concerned about air quality in the Gladstone Harbour area"		
"The water quality in Gladstone Harbour has not affected how often I use the area in the last 12 months"		

* Safety of harbour for public usage

	Rank (first to fourth)	Score (First=100)
"I would be happy to eat seafood caught in the Gladstone Harbour area"		
"I feel safe being in the Gladstone Harbour area at night"		
Measure: Number of oil and chemical spills in the harbour		
Measure: Number of marine safety incidents in the harbour		

Liveability and wellbeing objective

The next set of indicators relates to the Liveability and Wellbeing objective of the GHHP:

***Liveability and wellbeing**: Contribution of the harbour to liveability of Gladstone*

The objective has only one indicator, involving two questions:

Contribution of the harbour to liveability of Gladstone

- *Gladstone Harbour makes living in Gladstone a better experience*
- *I regularly participate in community events in the Gladstone Harbour area*

*Contribution of the harbour to liveability of Gladstone

	Rank (first to second)	Score (First=100)
"Gladstone Harbour makes living in Gladstone a better experience"		
"I regularly participate in community events in the Gladstone Harbour area"		

Some questions about you and your answers

The purpose of these questions is to gain an idea of how confident you are about your responses, which groups you think you represent (may be more than one); and how you found the survey questions.

***How confident are you about your responses relating to the different sub-objectives in each category?**

	Very unsure	Unsure	Neither sure nor unsure	Sure	Very sure
Cultural heritage?	◯	◯	◯	◯	◯
Sense of place?	◯	◯	◯	◯	◯
Harbour access?	◯	◯	◯	◯	◯
Harbour usability?	◯	◯	◯	◯	◯
Liveability and wellbeing?	◯	◯	◯	◯	◯

***How confident are you about your responses for each of the three approaches used?**

	Very unsure	Unsure	Neither sure nor unsure	Sure	Very sure
Ranking (First, second, etc)	◯	◯	◯	◯	◯
Scoring (1-100)	◯	◯	◯	◯	◯

Thank you

If you wish to be included in the random draw for the gift vouchers, please enter your details below:

Name

Email Address

Thank you for participating in this study.

For further information about the survey and how it will be used, please contact Sean Pascoe (sean.pascoe@csiro.au).

If you would like to make comments about the survey, please do so below.
