# Supplementary material for: Developing a Social, Cultural and Economic Report Card for a Regional Industrial Harbour
Source: PLoS One. 2016 Feb 3;11(2):e0148271. doi: 10.1371/journal.pone.0148271 (PMC4740459; doi:10.1371/journal.pone.0148271)
Supplement: S2 File — (PDF) [file pone.0148271.s002.pdf]

Developing a social, cultural and economic report card for a regional industrial harbour

Contents

1 Key demographic characteristics of the community survey sample 2

1.1 Full sample characteristics 2

1.2 Online survey (objective weightings) 4

2 BBN Sensitivity analysis 6

3 References 11

Figures

Figure A. The age structure of the population and sample (n=400) in the Gladstone Harbour region. 2

Figure B. Comparison of respondents versus non-respondents by a) age group, b) income group and c) gender. The horizontal line in each graph represents the overall average response rate. 5

Tables

Table A. Comparison of sample and population age distribution 3

Table B. Comparison of sample and census income distribution 3

Table C. Sensitivity analysis, Cultural component 8

Table D. Sensitivity analysis, Social sub-components 9

Table E. Sensitivity analysis, Economic component 10

1 Key demographic characteristics of the community survey sample

1.1 Full sample characteristics

The sample of survey respondents were divided evenly by gender (51% male, 49% female), and spread across all age group categories (Figure A), but fewer individuals were obtained from the younger age categories below that of 35 years (i.e. fewer within the following categories: 18-24 years and 25-34 years), and more from the older age categories (compared with the 2011 census distribution). Eleven percent of respondents identified themselves as Traditional Owners of the area.

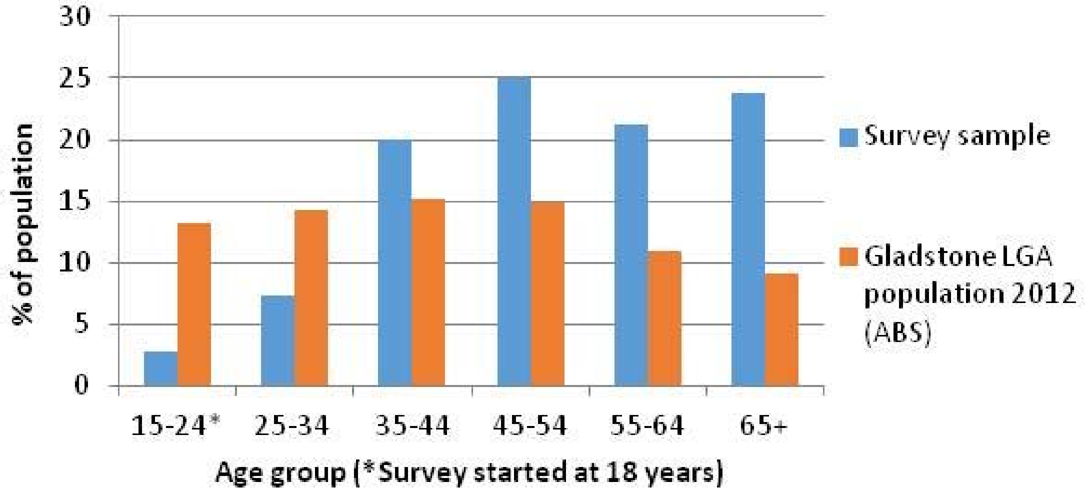

Figure A. The age structure of the population and sample (n=400) in the Gladstone Harbour region.

There is a significant difference between the sample and population in all age categories apart from the 33-44 years age group, see Table A.

Table A. Comparison of sample and population age distribution

Age	Survey	ABS census data	Sig. diff.
18 -24 years	3%	11%	*
25-34 years	7%	18%	*
35-44 years	20%	20%	
45-54 years	25%	19%	*
55-64 years	21%	13%	*
65 + years	24%	19%	*

* Indicates a statistical difference between the sample and the population when applying the normal approximation to the binomial test

Respondents were surveyed across all income categories. There were fewer individuals earning between $65,000-$78,000 and more in the highest income category (>$156,000).However, this was broadly consistent with the income distribution from the 2011 census (Table B).

Most respondents owned their home without a mortgage (41%) or with a mortgage (41%), rather than renting (14%). Those with a mortgage (n=163) typically did not pay greater than $3,000/month (83%). Of those that rent (n=55), most (78%) payed more than $175/week in rent. Respondents' homes typically contained 3 bedrooms (45% of respondents) or 4 bedrooms (39%). Most households (98%) owned a car.

Table B. Comparison of sample and census income distribution

Income	Survey	ABS census data	Sig. diff.
(Less than $20,799) Less than $399 weekly	12%	8%	*
($20,800-$41,599) $400 -$799 weekly	13%	13%	
($41,600-$64,999) $800 - $1249 weekly	10%	12%	
($65,000-$77,999) $1250 - $1499 weekly	5%	7%	
($78,000-$103,999) $1500 - $1999 weekly	18%	15%	
($104,000-$129,999) $2000 - $2499 weekly	12%	11%	
($130,000-$155,999) $2500 - $2999 weekly	11%	16%	*
($156,000+) $3000+ weekly	20%	20%	

* Indicates a statistical difference between the sample and the population when applying the normal approximation to the binomial test

Most (63%) of the survey respondents lived in the broader Gladstone urban area, while 14% lived in the southern beach areas of the Harbour. A further 20% lived outside the urban or beach areas, but within a radius of 20 km from the coast. The final 3% of respondents lived in other areas beyond 20km from the coast but within the broader Gladstone District.

1.2 Online survey (objective weightings)

The online survey was run over the period 11 September 2014 to 21 September 2014 using SurveyMonkey. A total of 228 initial invitations were sent out (based on those who agreed to participate from the CATI survey). Of these, 7 emails were incorrect (a transcription error during the CATI survey) and three people had previously opted out of SurveyMonkey surveys (so the email was not sent). As a result, the effective sample was 218 individuals.

In total, 83 responses to the community survey were received, representing a response rate of approximated 38%. With the exception of the 18-24 age group (none of which participated in the sub-components survey), the distribution of responses was fairly representative by age, income and gender (Figure B).

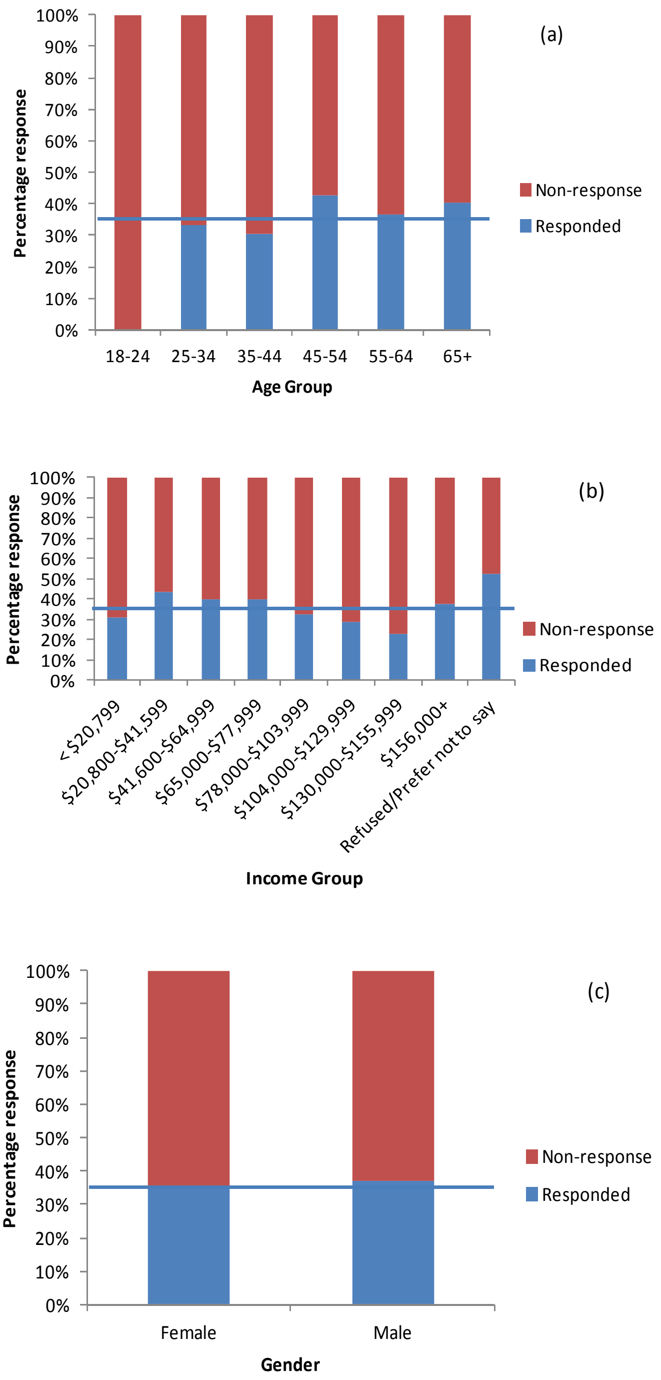

Figure B. Comparison of respondents versus non-respondents by a) age group, b) income group and c) gender. The horizontal line in each graph represents the overall average response rate.

2 BBN Sensitivity analysis

Sensitivity analysis can be used to measure the degree to which findings at any node (e.g. the indicator measure) can influence the outcomes (or beliefs) at another node (e.g. the sub-component value), given the set of findings currently entered. For the purposes of this study, it can indicate which indicators will be the most informative in determining the sub-component scores. The results are indicative only, as the sensitivity analysis considers only individual sensitivities – evidence in combination may have a larger impact that the "sum" of the individual impacts [1].

"Evidence" in BBNs is often uncertain in itself, and the cost of increasing the precision may be high. Sensitivity analysis can also be viewed as a means of determining which variables (indicators) require the most attention to get accurate data (or at least more precise assessments) as these will be the ones that the outcomes are most sensitive to [1].

Sensitivity analysis can also be used as part of the model evaluation. The sensitivity measures can be compared with a priori expectations about importance of particular nodes (indicators) to ensure that the model is behaving as expected [2]. If the plotted sensitivity function does not behave as expected, this may indicate errors in the network structure or the conditional probability tables (CPTs) [3].

Two forms of sensitivity analysis are commonly considered: mutual information (entropy reduction) and the expected reduction of real variance. Entropy relates to the uncertainty of a variable (*Q*) characterised by a probability distribution, $P(q)$ [3,4]. Entropy reduction reports the expected degree to which the joint probability of *Q* and *F* diverges from what it would be if *Q* were independent of *F*. That is, it is a measure of the mutual information shared between the two nodes. If $I(Q,\mathrm{F})$ is equal to zero, *Q* and *F* are mutually independent [3]

The mutual information (I) between Q and F is measured in "bits". The expected reduction in entropy of Q (measured in bits) due to a finding at F[1].

$$I = \sum_q \sum_f \log_2 \left[\frac{P(q)}{P(q)P(f)} \right] \tag{13}$$

where *q* is a state of the query variable (i.e. the sub-component) and *f* is a state of the varying variable (the indicator). The measure is logged with a base of 2, which is traditional for entropy and mutual information so that the units of the results will be "bits".

Variance Reduction refers to the expected reduction in variance of the expected real value of Q due to a finding at F.

$$Vr = \sum_q P(q) \left[X_q - \sum_q P(q) X_q \right]^2 - \sum_q P(q|f) \left[X_q - \sum_q P(q|f) X_q \right]^2 \tag{14}$$

where Xq is the numeric "real" value corresponding to state q (i.e. the sub-component). In this case, "real" refers to the expected value of continuous nodes, or discrete nodes which

[1] http://www.norsys.com/WebHelp/NETICA/X_Sensitivity_Equations.htm

have a real numeric value associated with each state. In our model, all nodes are continuous, with a value ranging from 0 (zero) to 1.

The results of the sensitivity analysis depend strongly on network parameters and on the current states of all observable nodes [5]. In our analysis, we assumed no prior information on the states of the nodes, with each state having an equal probability. The analysis hence assesses the effect on the sub-component node from moving from no information to full information (i.e. moving to either a zero or 100 percent likelihood of a state), given that no information (uninformed priors) exist in the other nodes not be adjusted.

The key sensitivity measures for the cultural performance component are presented in Table C. Three measures of sensitivity (variance reduction, mutual information and variance of beliefs, described in the methods section) are presented in order of decreasing sensitivity. That is, the higher the values, the more sensitive the overall result is to that measure, indicator or sub component. The Type column represents the type of Node variable, either sub-component (SC), indicator (I) or measure (M). A type of S also indicates it is derived from secondary data. Some Nodes have more than one type. A general rule of thumb is that variables that contribute less than 1% to variance reduction (or mutual information) are considered to be relatively uninformative individually. However, many of the measures that fall into this category relate to the indicators of values and attitudes towards Gladstone Harbour, which themselves are more influential on the outcome. Hence there is a cumulative effect of these measures that is not apparent from the individual effects.

The sensitivity measures for the social component are given in Table D. As with the cultural component, many measures have only a small individual impact on the overall outcome. The least sensitive measures are derived from secondary data, which are the most easily obtained. It is also likely that many of the measures below the 1% sensitivity level are unlikely to change substantially from one year to the next, so a lower frequency of data collection for these measures may be warranted.

The sensitivity measures for the social component are given in Table E. The outcome is most sensitive to recreational value, particularly land based recreational values. Employment, socio-economic status and commercial fishing have a lesser impact on the outcome. These are not likely to change substantially from one year to the next, so could be updated less frequently than other indicators and measures.

Table C. Sensitivity analysis, Cultural component

Node	Variance Reduction		Mutual Info		Variance of Beliefs	Type
	Measure	Percent	Measure	Percent		
Overall Cultural performance	0.01117	100.00	1.09780	100.00	0.25388	
Sense of Place	0.00510	45.60	0.37861	34.50	0.09199	SC
Cultural Heritage	0.00235	21.00	0.15695	14.30	0.02638	SC
Self efficacy	0.00115	10.30	0.07995	7.28	0.01822	I
Proportion of known indigenous sites protected	0.00069	6.13	0.04584	4.18	0.00940	I/M/S
Input into management	0.00052	4.61	0.03548	3.23	0.00779	M
Condition of non-indigenous heritage sites	0.00045	4.02	0.02865	2.61	0.00571	I/M/S
Quality of life	0.00042	3.79	0.02768	2.52	0.00661	M
Values of Gladstone Harbour	0.00039	3.52	0.02651	2.41	0.00588	I
Traditional sites protected	0.00027	2.44	0.01764	1.61	0.00365	I/M
Attitudes to Gladstone Harbour	0.00025	2.23	0.01657	1.51	0.00381	I
Measures of distinctiveness	0.00025	2.22	0.01582	1.44	0.00361	I
Self esteem	0.00024	2.15	0.01491	1.36	0.00367	I/M
Traditional owners consulted	0.00023	2.02	0.01451	1.32	0.00298	I/M
Continuity	0.00014	1.25	0.00898	0.82	0.00203	I
Who I am	0.00011	1.03	0.00730	0.67	0.00170	M
No place better	0.00009	0.80	0.00583	0.53	0.00132	M
Key part of community	0.00008	0.75	0.00548	0.50	0.00130	M
Stay 5 years	0.00007	0.63	0.00446	0.41	0.00103	M
Great asset to the region	0.00007	0.62	0.00453	0.41	0.00106	M
How long lived in the area	0.00005	0.47	0.00345	0.31	0.00076	M
Variety of marine life	0.00005	0.43	0.00317	0.29	0.00071	M
Great asset to Queensland	0.00005	0.41	0.00298	0.27	0.00069	M
Culturally special place	0.00004	0.40	0.00292	0.27	0.00066	M
Enjoy scenery and sights	0.00004	0.35	0.00259	0.24	0.00058	M
Spiritually special place	0.00004	0.34	0.00247	0.23	0.00056	M
Historical significance	0.00002	0.20	0.00146	0.13	0.00033	M
Attracts visitors to the region	0.00002	0.16	0.00116	0.11	0.00026	M
Opportunities for outdoor recreation	0.00002	0.15	0.00109	0.10	0.00025	M

Table D. Sensitivity analysis, Social sub-components

Node	Variance Reduction		Mutual Info		Variance of Beliefs	Type
	Measure	Percent	Measure	Percent		
Overall Social performance	0.01088	100.00	1.08715	100.00	0.26321	D
Liveability	0.00426	39.20	0.34942	32.10	0.08673	SC
Makes living a better experience	0.00209	19.20	0.15774	14.50	0.03582	M
Participate in community	0.00161	14.80	0.11607	10.70	0.03088	M
Harbour usability	0.00161	14.80	0.11799	10.90	0.02736	SC
Harbour access	0.00157	14.40	0.11173	10.30	0.03070	SC
Air and water quality	0.00054	4.96	0.03719	3.42	0.00954	I
Satisfaction with access	0.00039	3.59	0.02676	2.46	0.00723	I/M
Harbour safety	0.00030	2.74	0.02026	1.86	0.00514	I
Harbour health	0.00027	2.52	0.01857	1.71	0.00533	I
Satisfaction with Harbour	0.00025	2.32	0.01713	1.58	0.00414	I
Water quality does not affect access	0.00018	1.65	0.01216	1.12	0.00302	M
Barriers to access	0.00017	1.59	0.01173	1.08	0.00332	I
Satisfaction with ramps, etc	0.00017	1.53	0.01127	1.04	0.00319	I
How satisfied last trip?	0.00015	1.39	0.01022	0.94	0.00242	M
Water quality satisfaction	0.00015	1.34	0.00980	0.90	0.00265	M
Air quality satisfaction	0.00014	1.29	0.00945	0.87	0.00254	M
Happy to eat seafood	0.00012	1.13	0.00827	0.76	0.00213	M
Optimistic about future	0.00009	0.81	0.00594	0.55	0.00172	M
Great condition	0.00007	0.67	0.00491	0.45	0.00143	M
Improved over last 12 months	0.00007	0.67	0.00486	0.45	0.00141	M
Safe at night	0.00007	0.61	0.00449	0.41	0.00112	M
Quality of ramps and facilities	0.00006	0.59	0.00432	0.40	0.00110	M
Access to public spaces	0.00006	0.52	0.00378	0.35	0.00107	M
Shipping reduced use	0.00005	0.48	0.00350	0.32	0.00100	M
Frequency of use	0.00004	0.37	0.00267	0.25	0.00077	M
Number of ramps	0.00004	0.35	0.00252	0.23	0.00072	M
Marine debris affects access	0.00003	0.28	0.00203	0.19	0.00058	M
Marine debris a problem	0.00003	0.27	0.00200	0.18	0.00058	M
Recreational boats reduces access	0.00003	0.27	0.00196	0.18	0.00056	M
Marine safety incidents	0.00001	0.10	0.00075	0.07	0.00019	M/S
Oil spills	0.00001	0.09	0.00068	0.06	0.00018	M/S

Table E. Sensitivity analysis, Economic component

Node	Variance Reduction		Mutual Info		Variance of Beliefs	Type
	Measure	Percent	Measure	Percent		
Overall Economic performance	0.01048	100.00	1.03099	100.00	0.24658	
Recreational value	0.00461	44.00	0.38973	37.80	0.08980	SC
Land based recreation	0.00404	38.60	0.31982	31.00	0.07535	M
Direct economic footprint	0.00151	14.40	0.10818	10.50	0.03283	SC
Shipping activity	0.00151	14.40	0.10818	10.50	0.03283	I/M/S
Economic stimulus to community	0.00092	8.78	0.06352	6.16	0.01931	E
Recreational fishing	0.00009	0.89	0.00641	0.62	0.00188	I/M
Beach recreation	0.00006	0.61	0.00436	0.42	0.00129	I/M
Employment	0.00002	0.23	0.00162	0.15	0.00045	I/M/S
Socio-economic status	0.01048	100.00	1.03099	100.00	0.24658	I/M
Tourism occupancy	0.00461	44.00	0.38973	37.80	0.08980	I/M/S
Commercial fishing	0.00000	0.00	0.00000	0.00	0.00000	I/S
Trawl fisheries	0.00000	0.00	0.00000	0.00	0.00000	M/S
Pot fisheries	0.00000	0.00	0.00000	0.00	0.00000	M/S
Net fisheries	0.00000	0.00	0.00000	0.00	0.00000	M/S
Line fisheries	0.00000	0.00	0.00000	0.00	0.00000	M/S

3 References

1. Jensen FV, Nielsen TD (2007) Bayesian networks and decision graphs: Springer Verlag.
2. Chen SH, Pollino CA (2012) Good practice in Bayesian network modelling. Environmental Modelling & Software 37: 134-145.
3. Pollino CA, Woodberry O, Nicholson A, Korb K, Hart BT (2007) Parameterisation and evaluation of a Bayesian network for use in an ecological risk assessment. Environmental Modelling & Software 22: 1140-1152.
4. Korb KB, Nicholson AE (2003) Bayesian artificial intelligence: Chapman & Hall/CRC.
5. Bednarski M, Cholewa W, Frid W (2004) Identification of sensitivities in Bayesian networks. Engineering Applications of Artificial Intelligence 17: 327-335.
